# A Prevalence of Imprinted Genes within the Total Transcriptomes of Human Tissues and Cells

**DOI:** 10.1155/2012/793506

**Published:** 2012-09-11

**Authors:** Sergey V. Anisimov

**Affiliations:** ^1^Research Unit of Cellular and Genetic Engineering, V. A. Almazov Federal Center for Heart, Blood & Endocrinology, Akkuratova Street 2, Saint-Petersburg 197341, Russia; ^2^Department of Intracellular Signaling and Transport, Institute of Cytology, Russian Academy of Sciences, Tikhoretsky Prosp. 4, Saint-Petersburg 194064, Russia

## Abstract

Genomic imprinting is an epigenetic phenomenon that causes a differential expression of paternally and maternally inherited alleles of a subset of genes (the so-called imprinted genes). Imprinted genes are distributed throughout the genome and it is predicted that about 1% of the human genes may be imprinted. It is recognized that the allelic expression of imprinted genes varies between tissues and developmental stages. The current study represents the first attempt to estimate a prevalence of imprinted genes within the total human transcriptome. *In silico* analysis of the normalized expression profiles of a comprehensive panel of 173 established and candidate human imprinted genes was performed, in 492 publicly available SAGE libraries. The latter represent human cell and tissue samples in a variety of physiological and pathological conditions. Variations in the prevalence of imprinted genes within the total transcriptomes (ranging from 0.08% to 4.36%) and expression profiles of the individual imprinted genes are assessed. This paper thus provides a useful reference on the size of the imprinted transcriptome and expression of the individual imprinted genes.

## 1. Introduction

Genomic imprinting is an epigenetic phenomenon that causes a differential expression of paternally and maternally inherited alleles of a minor subset of genes (the so-called imprinted genes). Genomic imprinting was first discovered in 1984 [1, 2], and in 1991 the first imprinted genes (IGF2, paternally expressed; IGF2R and H19, maternally expressed) were identified in the mouse [3–5]. Since then, the imprinting status was confirmed for numerous genes in *Homo sapiens *and *Mus musculus *genomes, less for *Bos taurus*, *Rattus norvegicus*, *Sus scrofa*, *Canis lupus familiaris,* and *Ovis aries*; many more genes are considered candidates [6]. Functional significance of the genomic imprinting is not yet fully understood [7–9], while alterations in the expression of imprinted genes are linked to certain pathologies, including Angelman syndrome, Prader-Willi syndrome, and particular cancer subtypes. Genomic imprinting varies between species and tissues. Furthermore, it is a dynamic process and may vary depending on the developmental stage [10]. The goal of the study was to estimate a prevalence of imprinted genes within the total human transcriptome, in cell and tissue samples in a variety of physiological and pathological conditions.

Serial analysis of gene expression (SAGE) is a sequence-based technique to study mRNA transcripts quantitatively in cell populations [11]. Two major principles underline SAGE: first, short (10 bp) expressed sequenced tags (ESTs) are sufficient to identify individual gene products, and second, multiple tags can be concatenated and identified by sequence analysis. SAGE results are reported in either absolute or relative numbers of tags, which permits direct comparisons between tag catalogues and datasets [12–15]. Numerous technical adaptations assured a development of similar techniques [16], yet SAGE remains an important tool of modern molecular biology. It is widely used in a number of applications, of which a molecular dissection of cancer genome is the major [17]. In the current study, expression of established and candidate imprinted genes was evaluated in a wide array of cell and tissue samples using a comprehensive set of currently available SAGE data for *Homo sapiens*. Five hundred eighty-one SAGE catalogues based on the libraries generated with most commonly used *NlaIII* anchoring enzyme were screened using a conservative set of criteria, and in 492 of these (accounting for nearly 36 million SAGE tags) gene expression profiles of the imprinted genes were analyzed, using a proved algorithm [18]. It was therefore possible to estimate a prevalence of imprinted genes within the total human transcriptome.

## 2. Methods

### 2.1. Imprinted Gene Subsets

 Established and candidate imprinted gene subset was assembled based on the Geneimprint resource (http://www.geneimprint.com/; credits to R.L. Jirtle) and Luedi et al. study [6]. Of the latter, high-confidence imprinted human gene candidates predicted to be imprinted by both the linear and RBF kernel classifiers learned by Equbits Foresight and by SMLR ([6], supplementary data) were utilized. Redundant entries have been excluded.

### 2.2. SAGE

 SAGE technology is based on isolation of short tags form the appropriate position within the mRNA molecule, followed by the concatemerization of the tags, sequencing, tag extraction and gene annotation [11]. The complete set of publicly available SAGE libraries (GPL4 dataset, *NlaIII* anchoring enzyme) was downloaded from the Gene Expression Omnibus (GEO) database (National Center of Biotechnology Information (NCBI); http://www.ncbi.nlm.nih.gov/geo/). Following an exclusion of the duplicate entries, SAGE libraries were annotated and sorted based on the number of tags sequenced. Noninformative (A)_10_ sequences were extracted from SAGE libraries when detected, and tags per million (tpm) values were recalculated accordingly for all libraries as the transcript's raw tag count divided by the number of reliable tags in the library and multiplied by 1,000,000. SAGE libraries, constructed by Potapova et al. [19], were a subject to a “clean-up” procedure through which all clones containing ≤4 tags were excluded [20], with the remaining tags constituting the pool of “reliable tags.”

### 2.3. SAGE Tag Annotation

Established and candidate imprinted gene subset has matched CGAP (Cancer Genome Anatomy Project, NCI, NIH) SAGE Anatomic Viewer (SAV) applet [17]. For genes not matching SAV applet entries, and when unreliable/internal tags were suggested by SAV applet (viz., for TIGD1, HOXA3, NTRI genes, etc.), reliable 3′ end tags were extracted from full-length sequences available via GenBank (NCBI, NIH).

### 2.4. Expression Profiling

SAGE tags was matched the individual SAGE catalogues using MS Access software package Query function. Individual queries (both absolute tag abundance per library and normalized tag per million (tpm) values) were merged using MS Excel software. Calculations of maximal and average expression of transcripts matching established and candidate imprinted genes were performed using normalized tpm values. Particular values could be recalculated to the fraction of the total gene expression by dividing tpm value by 1,000,000.

### 2.5. Clustering Analysis

Clustering analysis was performed using EPCLUST Expression Profile data CLUSTering and analysis software (http://www.bioinf.ebc.ee/EP/EP/EPCLUST/). K-mean clustering analysis was performed after transposing the data matrix with initial clusters chosen by most distant (average) transcripts. For each dataset, the number of clusters was set to the lowest value yielding one cluster containing a solitary database entry. Hierarchical clustering was performed using correlation measure-based distance/average linkage (average distance) clustering method; hierarchical trees were built for individual datasets.

## 3. Results

 Established and candidate human imprinted gene subset (203 entries total) was assembled based on the Geneimprint resource and Luedi et al. study data [6]. Of the candidate imprinted genes identified in the latter, high-confidence gene candidates (predicted via Equbits Foresight and SMLR means [6]) were selected. Following exclusion of the redundant entries, appropriate short (10 bp) SAGE tags matching *NlaIII* anchoring enzyme were annotated to gene targets using CGAP (Cancer Genome Anatomy Project, NCI, NIH) SAGE Anatomic Viewer (SAV) applet or manually, as described earlier [18]. For a number of the candidate imprinted genes, a complete sequence was unavailable via GenBank or alternative databases (e.g., GenBank ID: NM_016158, NM_024547, NM_181648, etc.), for that reason, a volume of the human imprinted gene subset subjected to tag annotation was reduced to 174 genes. Of these genes, candidate imprinted gene Q9NYI9 (PPARL; GenBank ID: AF242527) could not be annotated with SAGE tag, missing *NlaIII* anchoring enzyme recognition sites completely. Therefore, a total of 173 genes (including 53 established imprinted genes and 120 candidate imprinted genes) were annotated with the appropriate SAGE tags (Table 1) and subjected to further analysis.

The complete set of publicly available human SAGE catalogues was downloaded from the Gene Expression Omnibus (GEO, NCBI) database. Acquired SAGE catalogues represent 581 SAGE libraries generated from a wide spectrum of cell and tissue samples in a variety of physiological and pathological conditions. Following an exclusion of the numerous duplicate GEO database entries (e.g., GSM785 = GSM383907; GSM1515 = GSM383958; GSM85612 = GSM125353, etc.), the criteria listed below were applied when selecting libraries for the analysis of gene expression. SAGE libraries were selected only if they have represented (i) genetically unaltered/unmodified samples, (ii) SAGE catalogues with a total number of tags ≥20,000, and (iii) a complete dataset available. For example, samples GSM383929 and GSM180669 were excluded since these did not satisfy criteria (i), representing ovary surface epithelium immortalized with SV40 and lymphocytes from Down syndrome children, respectively; samples GSM384024 (white blood cells, CD45^+^, isolated from a mammary gland carcinoma; 18,741 tags) and GSM1128 (breast cancer cell line; tags detected once are not available) were excluded as not satisfying criteria(ii) and (iii), respectively (Supplementary Table 1). Due to the conservative nature of the criteria listed above, a total number of SAGE catalogues satisfying these and thus selected for further analysis (i.e., to the extraction of tags matching imprinted genes) was reduced to 492. Together, these 492 SAGE catalogues representing human samples account for 35.97 million SAGE tags constructed using *NlaIII-*anchoring enzyme. The catalogues were assigned into one of the following Clusters: C (cancer tissue; 185 SAGE catalogues), N (normal tissue and cells; 166 SAGE catalogues), IV (cells cultured *in vitro*; 112 SAGE catalogues), or D (nontumorous disease tissue and cells; 29 SAGE catalogues) (Table 2, and Supplementary Table 1).

**Figure 1:**
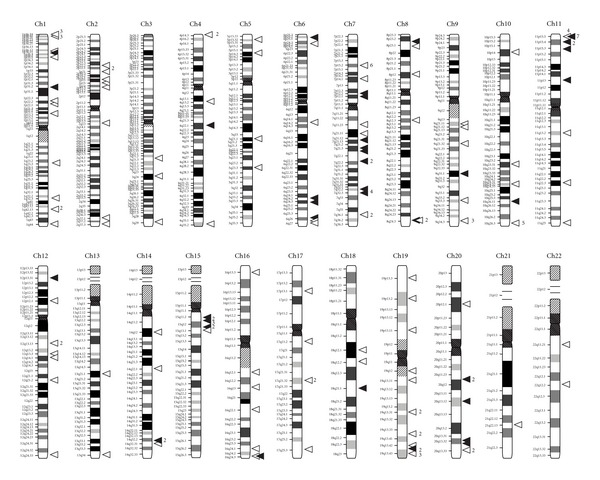
A schematic representation of the analyzed established (53, filled arrowheads) and candidate (120, empty arrowheads) imprinted genes distribution through the human genome. Chromosome layout is via NCBI (Build 37.2). Numbers next to some of the arrowheads indicate the number of entries per locus.

**Figure 2:**
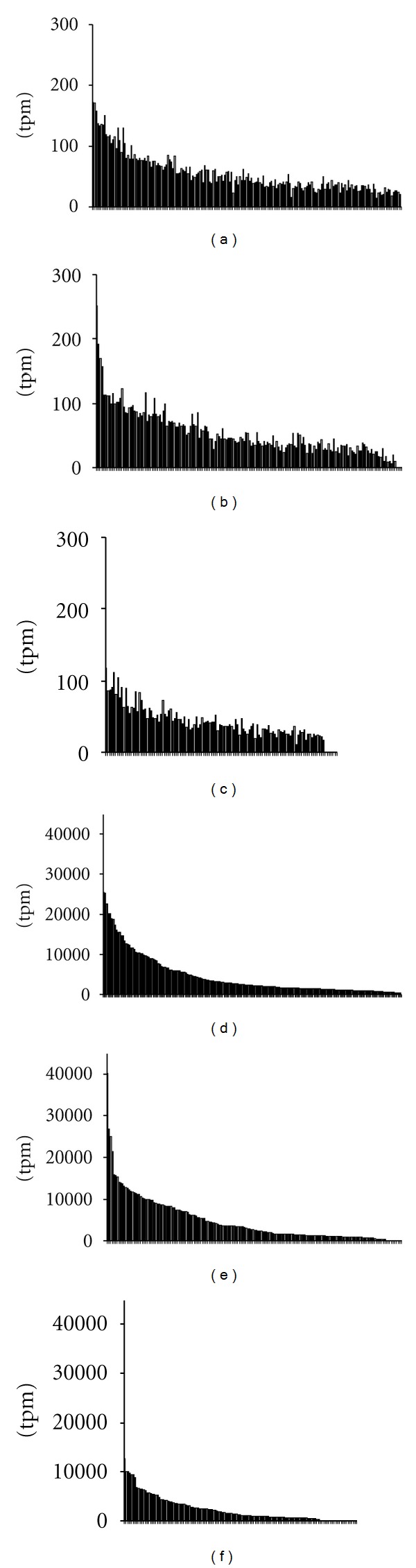
Histogram of average ((a), (b), and (c)) and maximum ((d), (e), and (f)) tag per million (tpm) values of the pool of imprinted genes and gene candidates for the normalized SAGE catalogues: cancer tissue ((a), (d); 185 catalogues); normal tissues and cells ((b), (e); 166 catalogues); cells cultured *in vitro* ((c), (f); 112 catalogues). Corresponding histogram pairs are built following a sorting by the maximum value in the pool.

Figure 1 shows a distribution of the analyzed established and candidate imprinted genes through the human genome. Primary analysis of the normalized expression profiles of the imprinted genes demonstrated a great variability in the cumulative gene expression for 173 genes (Figure 2, Table 3, and Supplementary Table 2). Average cumulative gene expression of those genes in human tissues and cells was 0.90% of the total gene expression: specifically, 0.95% for both cancer and normal tissue and cells (clusters C and N, resp.), 0.77% for cells cultured *in vitro* (cluster IV), and 0.83% for nontumorous disease tissue and cells (cluster D). In the pool of the assessed SAGE catalogues, it ranged from 0.08% (total blood, GSM389907 [21]) to 4.36% of the total gene expression (bronchial epithelium, GSM125353 [22]). Of 492 human SAGE catalogues tested, the cumulative expression of the imprinted genes constituted >2% of the total gene expression in 21 and <0.2% in 7 catalogues. The SAGE libraries with 10% most and 10% least cumulative and average expression of established and candidate imprinted gene subsets are listed in Table 3.

In some samples, a major fraction of the cumulative expression of the imprinted genes was established by only a few highly abundant transcripts. For example, in the GSM125353 SAGE catalogue already mentioned above, 91.9% of the cumulative (total) gene expression of the assayed imprinted genes is represented by the single gene, namely, PTPN14 (ACTTTTTCAA tag). Similarly, in GSM383893 SAGE catalogue (gallbladder tubular adenocarcinoma [17, 23]), the same gene constitutes 86.6% of the cumulative (total) gene expression of the assayed imprinted genes. In many other SAGE catalogues, expression profile of the assayed imprinted genes was rather more balanced. For example, in the GSM383840 SAGE catalogue (mammary myoepithelium, CD10^+^ cells [24]), PTPN14 constitutes just 8.7% of the cumulative (total) gene expression of the assayed imprinted genes, equal to GNAS gene (ATTAACAAAG tag). Some imprinted genes were expressed almost ubiquitously through the samples: for example, genes NDUFA4, RPL22, Q8NE65, PTPN14, GNAS, and RAB1B (Supplementary Table 3). Notably, in other cases, expression of the particular imprinted genes either was not detected at all in all 492 SAGE catalogues screened (EVX1, ACGCCCGTGG tag), or was detected only occasionally (Supplementary Table 3). For example, gene DUX2 (AAGGGGTGGA tag) expression was detected only 3 times (on a minimum level) in 492 SAGE catalogues representing cell and tissue samples in a variety of physiological and pathological conditions: namely, in GSM383692 SAGE catalogue (astrocytoma grade II [25]), GSM383867 SAGE catalogue (colon carcinoma cell line [17, 23]), and GSM383928 SAGE catalogue (ovary preneoplasia cell line [26]). Similarly rare was the expression of FAM75D1 (detected only 3 times altogether), FAM77D, ISM1, FLJ20464, and Q8NB05 (detected only 5 times, in all cases on a minimum level).

**Figure 3:**
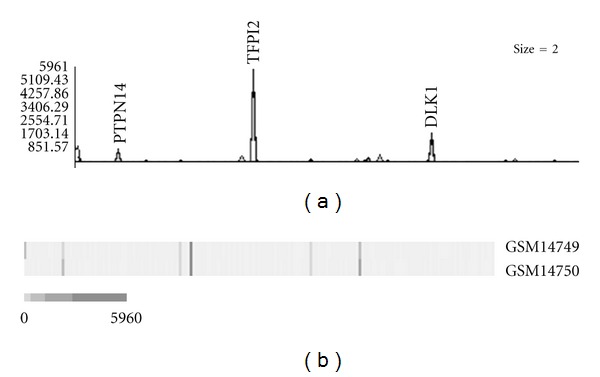
An example of gene expression pattern recognized by K-mean clustering analysis (normal tissue and cells, cluster 5). Graph line (a) and cluster contents (b). Vertical bars denote individual genes. Exponential shades of grey code (5 colors) are based on the normalized tpm values. GSM14749: first trimester placenta; GSM14750: full-term placenta. Imprinted genes with peak expression values in the cluster are indicated. PTPN14: protein tyrosine phosphatase, nonreceptor type 14; TFPI2: tissue factor pathway inhibitor 2; DLK1: delta-like 1 homolog (Drosophila).

**Figure 4:**
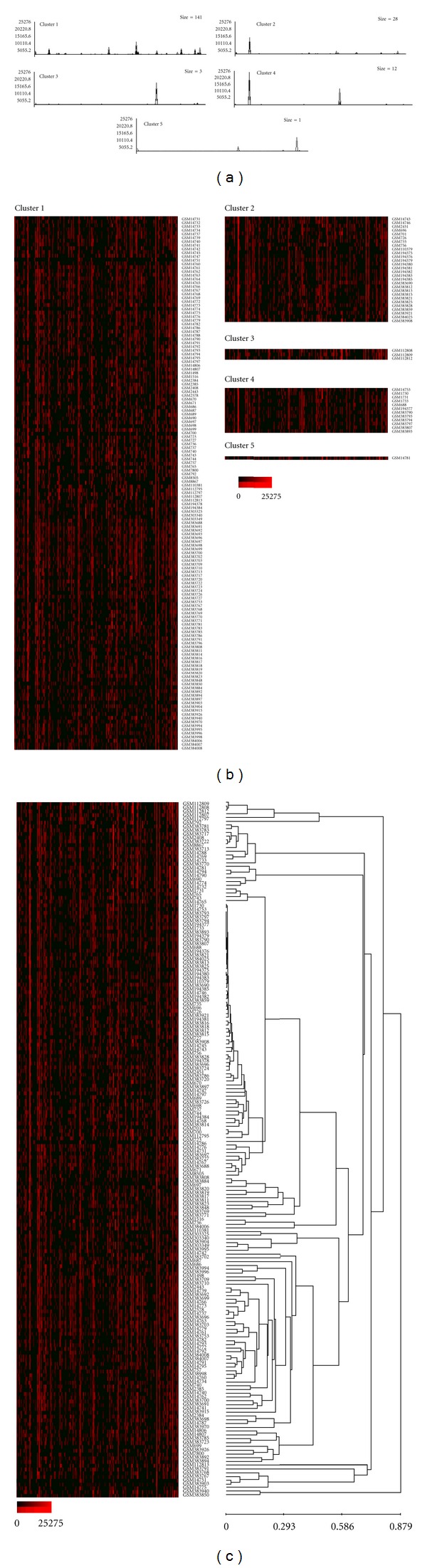
Gene expression patterns recognized by K-mean and hierarchical clustering analysis (cancer tissue, 185 SAGE catalogues). K-mean clustering analysis, (a) graph lines and (b) cluster contents; vertical bars denote individual genes. (c) Hierarchical cluster tree. Exponential shades of red code (15 colors) are based on the normalized tpm values.

**Figure 5:**
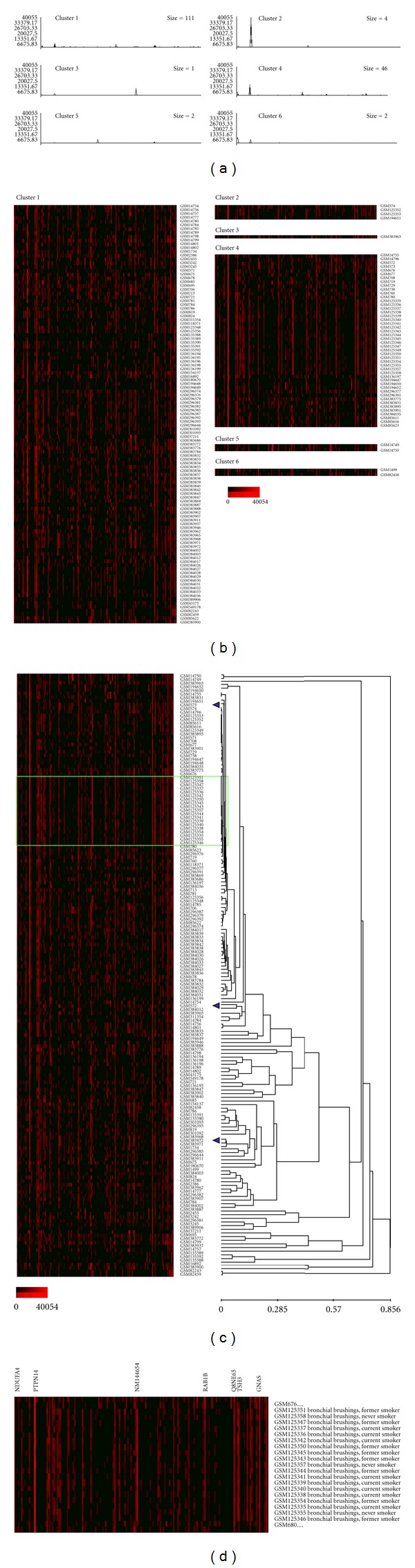
Gene expression patterns recognized by K-mean and hierarchical clustering analysis (normal tissue and cells, 166 SAGE catalogues). K-mean clustering analysis, (a) graph lines and (b) cluster contents; vertical bars denote individual genes. Arrowheads point out 3 SAGE libraries generated from peripheral retinal samples. (c) Hierarchical cluster tree, fragment (d) enlarged is highlighted. Exponential shades of red code (15 colors) are based on the normalized tpm values.

**Figure 6:**
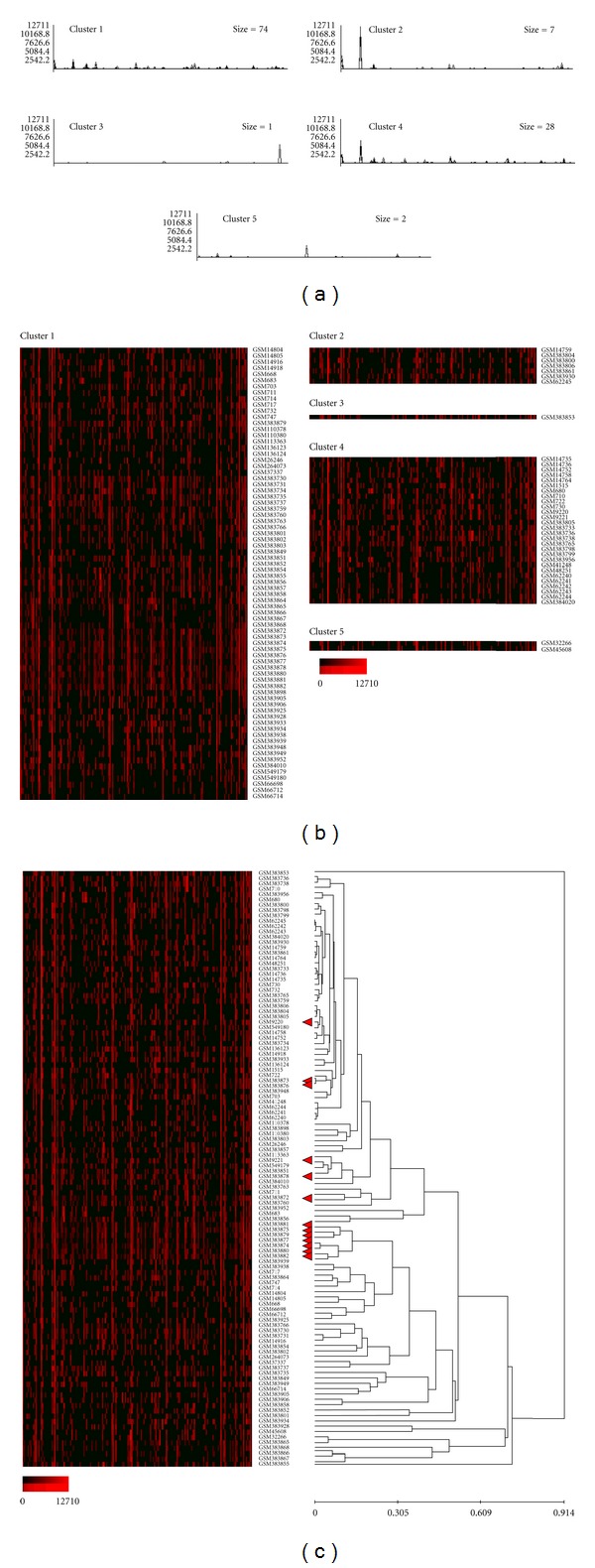
Gene expression patterns recognized by K-mean and hierarchical clustering analysis (cells cultured *in vitro*, 112 SAGE catalogues). (a) K-mean clustering analysis, graph-lines and (b) cluster contents; vertical bars denote individual genes. (c) Hierarchical cluster tree. Arrowheads point out 13 SAGE libraries generated from undifferentiated embryonic stem cells (ESC). Exponential shades of red code (15 colors) are based on the normalized tpm values.

**Figure 7:**
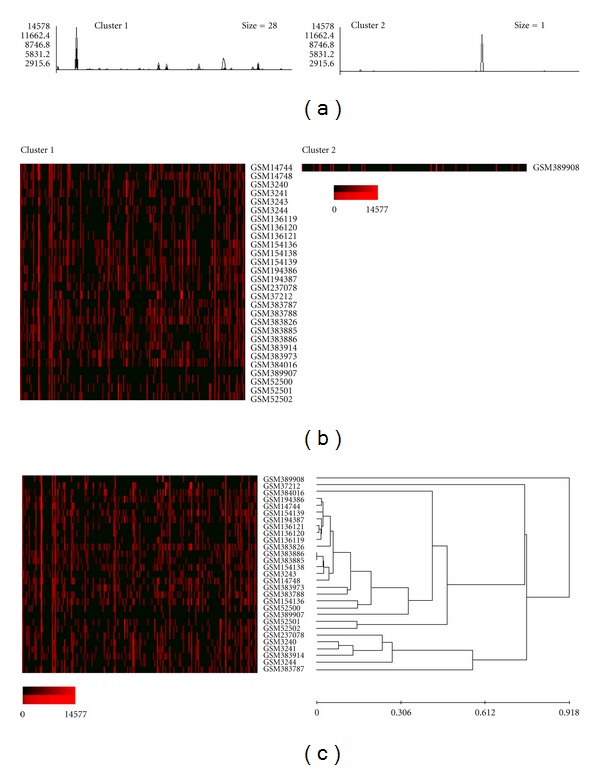
Gene expression patterns recognized by K-mean and hierarchical clustering analysis (nontumorous disease tissue and cells, 29 SAGE catalogues). (a) K-mean clustering analysis, graph lines and (b) cluster contents; vertical bars denote individual genes. (c) Hierarchical cluster tree. Exponential shades of red code (15 colors) are based on the normalized tpm values.

To assess variation in the expression of individual imprinted genes in the samples, the clustering analysis of the normalized expression profiles was performed using EPCLUST (Expression Profile data CLUSTering and analysis) software. For each dataset, the number of clusters was set to the lowest value yielding one cluster containing a solitary database entry; 5 for cancer tissue, 6 for normal tissues and cells, 5 for cells cultured *in vitro*, and 2 for nontumorous disease tissue and cells (Figures 3–7). Notable diversity was observed in the transcription profiles represented by the individual clusters, with relatively high expression levels characteristic for just 1-2 or a higher number of the individual imprinted genes (Figures 4(a), 4(b)–7(a), and 7(b)). Expectedly, in a few cases samples generated from the same tissues/cell types did fell into the same compact cluster of the distinct pattern (Figure 3, Figures 4(a), 4(b)–7(a), and 7(b)). However, in many other cases imprinted gene expression profiles of the same/similar tissue or cell types fall into different clusters. Similarly, though in many cases imprinted gene expression profiles of the same/similar tissue or cell types fell into the closely matching area of the hierarchical tree built for the individual datasets (clusters C, N, IV, and D) (Figures 4(c), 5(c), 5(d), 6(c), and 7(c)), in other cases notable variability was observed in the distribution of imprinted gene expression profiles of the same/similar tissue or cell types. For example, at K-mean clustering, small-size cluster 3 in cancer tissue dataset (3 entries) is composed entirely of neuroblastoma samples (Figures 4(a) and 4(b)); however, other entries representing tumors of the same histological properties [27] fell into cluster 1 (composed of 141 entries in total). Cluster 4 in the same dataset (12 entries) is composed entirely of carcinoma samples, while cluster 2 (28 entries) is composed of carcinoma samples predominantly (19 entries), with other samples representing astrocytoma (3 entries), glioblastoma multiforme (2 entries), cystadenoma (1 entry), rhabdosarcoma (1 entry), and unclassified breast cancer (2 entries). Similarly, only one cluster in the normal tissue and cell dataset has a homogenous composition (cluster 5, 2 entries), matching both available SAGE libraries constructed from placenta (GSM14849, also designated GSM383945; GSM14750, also designated GSM383947 [17]) (Figure 3), with all other clusters composed of the samples of diverse origins. Illustratively, this particular cluster brakes down (i.e., cluster content get redistributed to the clusters of the smaller size) only if the number of K-mean clusters for the dataset is increased from the set value of 6 to 26, while some other clusters break down more readily. In the hierarchical trees, most densely packed areas (representing most similar transcription profiles) are generally composed of the samples of the same/similar tissue or cell types. For example, one of the densest areas in four hierarchical trees built is composed of 19 samples matching bronchial brushings (Figures 5(c) and 5(d)) [22], with all 5 other samples of the same origin falling into the nearest vicinity within the hierarchical tree (Figure 5(c)). At the same time, some SAGE libraries representing the samples of the identical origin fell into the separate K-mean clusters and into well-separated areas of the hierarchical tree. This was observed, for example, for 3 available peripheral retina samples, from which GSM572 and GSM573 [28] fell into cluster 3, and GSM383968 [29] fells into cluster 1 (Figure 4).

## 4. Discussion

Mechanism of genomic imprinting plays important, yet not fully understood role in many physiological processes: in particular, in the control of growth and development. Since the identification of the first imprinted genes (IGF2, IGF2R, and H19) in mouse in 1991, a large volume of information has been accumulated on the identity and biological function of imprinted genes both for *Homo sapiens* and animal species (*Mus musculus* in particular). Over the course of the decade, we witness an expansion of the list of the established imprinted genes [6, 30]. It is most probable that novel candidate imprinted genes will be identified in the future, and features of the imprinted genes will be confirmed for some candidates. In the current study, a comprehensive list of the human imprinted genes and high-confidence gene candidates (203 entries total) became a subject for a large-scale *in silico* gene expression profiling. Available nucleotide sequences (174 genes and gene candidates) have been utilized for the extraction of the appropriate short SAGE tags matching *NlaIII* anchoring enzyme, most common in generating SAGE libraries. Notably, candidate imprinted gene Q9NYI9 (PPARL) did not bear *NlaIII* recognition sites. This limitation of the conventional SAGE protocol can generally be overcome by using an alternative anchoring enzyme [16]. However, gene Q9NYI9 does not bear recognition sites for anchoring enzymes *Sau3AI *and* RsaI *(second and third most common in generating SAGE libraries) as well, though it bears one for *MmeI *utilized in LongSAGE protocol. Taken together, not 174 but 173 genes (missing Q9NYI9 (PPARL))—including 53 established imprinted genes and 120 candidate imprinted genes—were annotated with the appropriate SAGE tags. The latter was matched the pool of 492 normalized SAGE catalogues representing libraries derived from human samples, constructed using *NlaIII *anchoring enzyme and together accounting for 35.97 million SAGE tags. Collectively, these catalogues represent a comprehensive assay of tissues and cell types in physiological and a variety of pathological conditions. Gene expression of imprinted genes was assessed in the normalized SAGE catalogues representing the transcriptomes of these samples, according to the straightforward algorithm of *in silico* analysis.

As with nearly any other gene, expression of imprinted genes is not a constant, but rather a dynamic function of cell type and state. In the current study, a great variability was observed in both cumulative/total expression of the studied imprinted genes and that of the individual genes. The cumulative expression of 173 studied imprinted genes ranges from 0.08% (total blood) to 4.36% (bronchial epithelium) of the total gene expression (Table 3). In some samples (Table 3 and Supplementary Table 2), imprinted genes-associated proportion of the transcriptome is obviously above what is to be expected from such a limited group of genes, clearly reflecting the importance of the biological roles played by the latter. At the same time, overall expression of the imprinted genes was equal in the clusters of cancer tissues and normal tissue and cells (clusters C and N, 0.95% for both clusters) and lower for the cells cultured *in vitro* (cluster IV, 0.77%).

The current study apparently represents the first attempt to estimate an impact of imprinted genes on the total volume of the transcriptome. Obvious biases affect an accuracy of the algorithm applied, suggesting both underestimation (probable existence of yet unidentified imprinted genes, unavailable information on gene structure for some imprinted genes, absence of anchoring enzyme recognition sites for at least one gene) and overestimation (unconfirmed imprinting status of some of the candidate imprinted genes, SAGE tags matching more than one gene; see Table 1) of the relative size of the imprinted transcriptome. Despite this, provided data on the estimated cumulative/total expression of the known imprinted genes (their number well corresponding to the predicted number of imprinted genes in human genome [31, 32]) in a variety of tissues and cells is most interesting. Until now, little information was available on the overall expression of imprinted genes in the cells of different types. It is generally believed that many imprinted genes are highly expressed in the developing and adult brain tissue [33], placenta [34], and undifferentiated stem cells [35]. Discrete studies identify certain highly expressed imprinted genes as the potential biomarkers of cancer subtypes [36, 37]. In contrast, imprinted genes are known to be expressed on relatively low level in adult blood cells [38]. This information is supported by the observed values of the cumulative expression of the imprinted genes through the screened samples (Table 3 and Supplementary Table 2): cumulative expression of the imprinted genes is generally high in many assessed brain-derived samples and low in blood samples. It was also observed earlier that major upregulation of gene expression of the numerous imprinted genes is associated with early differentiation and development, rather than with undifferentiated status of stem cells [39, 40]. Concordantly, in the current study, all of the 13 SAGE libraries generated from undifferentiated embryonic stem cells (ESCs)—namely, lines HES3, HES4 [17, 23, 41], BG01, H1, H7, H9, H13, H14, HSF6 [17, 23]—uniformly demonstrate intermediate cumulative expression of the imprinted genes (Supplementary Table 2) and fit closely in the hierarchical tree built for the corresponding cluster (cluster IV; Figure 5(c)). However, many samples with high cumulative expression of the imprinted genes do not fit into any of the groups listed above. Important role of genomic imprinting in particular normal cell and cancer subtypes, suggested by high expression of these genes, thus should be a subject of the follow-up studies. Expression of individual imprinted genes varies to even further extent in the samples screened. Expression of the candidate imprinted gene even-skipped homeobox 1 (EVX1) was not detected in any sample submitted to the analysis, while the expression of many more (DUX2, FAM75D1, Q8NB05, FLJ20464, ISM1, FAM77D, and others) was detected only in a few samples, always on a minimal level. In contrast, further imprinted genes (NDUFA4, RPL22, Q8NE65, GNAS, PTPN14, RAB1B, and others) were expressed in the majority of the samples screened, often on high level (Supplementary Table 3).

Illustratively, a notable variation in the cumulative expression of the imprinted genes and in the expression of individual imprinted genes is observed in the cells cultured *in vitro*, including cells of the same type (e.g., numerous medulloblastoma, glioblastoma multiforme, and breast carcinoma cell lines) (Supplementary Table 2 and Figure 6). This observation further supports earlier suggestion that cell culture conditions contribute to the maintenance or alteration of the imprinted gene expression [42, 43].

Taken together, a screening of the normalized expression profiles of a comprehensive panel of the established and candidate imprinted genes within the publicly available human SAGE datasets was performed in the current study: the first to estimate a prevalence of imprinted genes within the total human transcriptome in a large scale. This paper thus provides a useful reference on the relative size of the imprinted transcriptome and on the expression of the individual imprinted genes.

## Supplementary Material

Supplementary Material provides key properties of established and candidate imprinted gene subset within the SAGE datasets.

## Figures and Tables

**Table 1:** SAGE tag annotation for established and candidate imprinted gene subset.

*N*	Gene symbol	Gene name	Aliases	Location^a^	Status	Expressed allele	*NlaIII* tag	Notes	GenBank accession number
1	NDUFA4	NADH dehydrogenase (ubiquinone) 1 alpha subcomplex, 4, 9 kDa		1p13.3	Candidate	Paternal	TTGGAGATCT		BC105295
2	GFI1	Growth-factor-independent 1 transcription repressor	ZNF163	1p22.1	Candidate	Paternal	TGTACCATAG		NM_001127215
3	NM019610	RNA-binding motif protein, X-linked-like 1 (RBMXL1), transcript variant 2		1p22.2	Candidate	Maternal	GCAGATTTAT		NM_019610
4	DIRAS3	DIRAS family, GTP-binding RAS-like 3	ARHI, NOEY2	1p31	Imprinted	Paternal	CAGAAAAAAA	* ^b^	BC005362
5	BMP8B	Bone morphogenetic protein 8b	OP2, BMP8, MGC131757	1p35–p32	Candidate	Paternal	AGCAAAACTG	*	NM_001720
6	FUCA1	Fucosidase, alpha-L- 1, tissue	FUCA	1p36.11	Candidate	Paternal	CTATTTAGTT		NM_000147
7	TP73	TP73	P73	1p36.3	Imprinted	Maternal	TGGTACCGCC		NM_001126240
8	PRDM16	PR domain containing 16	MEL1	1p36.32	Candidate	Paternal	AGATTGATAT		NM_022114
9	PEX10	Peroxisomal biogenesis factor 10		1p36.32	Candidate	Maternal	GGAGGCGGCG		NM_002617
10	WDR8	WD repeat domain 8		1p36.32	Candidate	Maternal	TCGGTGCAGG		NM_017818
11	DVL1	Dishevelled, dsh homolog 1 (*Drosophila*)	DVL	1p36.33	Candidate	Maternal	GCCCGCAGGG		NM_004421
12	Q5EBL5	Family with sequence similarity 132, member A	FAM132A	1p36.33	Candidate	Maternal	GTTTCCAGGC		NM_001014980
13	TMEM52	Transmembrane protein 52		1p36.33	Candidate	Paternal	TTACACCGGC		NM_178545
14	HSPA6	Heat shock 70 kDa protein 6 (HSP70B′)		1q23.3	Candidate	Maternal	TATGAATTTT		NM_002155
15	PTPN14	Protein tyrosine phosphatase, nonreceptor type 14	PEZ	1q32.3	Candidate	Maternal	ACTTTTTCAA	*	BC017300
16	HIST3H2BB	Histone cluster 3, H2bb		1q42.13	Candidate	Maternal	AACTCCTTCG	*#^c^	NM_175055
17	OBSCN	Obscurin, cytoskeletal calmodulin and titin-interacting RhoGEF	KIAA1556, KIAA1639	1q42.13	Candidate	Paternal	CTGAGCGCCG	*	NM_001098623
18	Q8NGX0	Olfactory receptor, family 11, subfamily L, member 1	OR11L1	1q44	Candidate	Paternal	AGAAGGAAAT	*	NM_001001959
19	VAX2	Ventral anterior homeobox 2	DRES93	2p13.3	Candidate	Maternal	GGCGATGGGG		NM_012476
20	OTX1	Orthodenticle homeobox 1		2p15	Candidate	Maternal	GCGGTTCCAG		BC007621
21	Q96PX6	Coiled-coil domain containing 85A	CCDC85A, KIAA1912	2p16.1	Candidate	Paternal	GCAGATATTC	R^d^	NM_001080433
22	ABCG8	ATP-binding cassette, subfamily G (WHITE), member 8		2p21	Candidate	Maternal	GGCTCCAAAA		NM_022437
23	ZFP36L2	Zinc finger protein 36, C3H type-like 2	ERF2, TIS11D	2p21	Candidate	Maternal	TAGAAAGGCA		NM_006887
24	CYP1B1	Cytochrome P450, family 1, subfamily B, polypeptide 1	P4501B1	2p22.2	Candidate	Paternal	AATGCTTTTA	*	NM_000104
25	RPL22	Ribosomal protein L22	EAP	2q13	Candidate	Paternal	GATGCTGCCA	*	CR456873
26	TIGD1	Tigger transposable element derived 1	EEYORE	2q37.1	Candidate	Paternal	CGAAAAGCTT	R	BC063500
27	MYEOV2	Myeloma overexpressed 2		2q37.3	Candidate	Paternal	CAGACTTTTT	*	AF487338
28	FTHFD	10-Formyltetrahydrofolate dehydrogenase	ALDH1L1; DKFZp781N0997	3q21.3	Candidate	Maternal	TCTGCATCTT		BC027241
29	ZIC1	Zic family member 1 (odd-paired homolog, *Drosophila*)	ZIC, ZNF201	3q24	Candidate	Maternal	ATAATAGTGG		NM_003412
30	HES1	Hairy and enhancer of split 1, (*Drosophila*)	HHL, HRY, HES-1, bHLHb39, FLJ20408	3q29	Candidate	Paternal	CACTATATTT		NM_005524
31	FGFRL1	Fibroblast growth factor receptor-like 1	FHFR, FGFR5	4p16.3	Candidate	Maternal	AAAGTGCATC		NM_001004358
32	SPON2	Spondin 2, extracellular matrix protein	DIL1	4p16.3	Candidate	Paternal	TTATGGATCT		NM_001128325
33	Q9NYJ6	Immunoglobulin superfamily, member 9	IGSF9, 644ETD8, Dasm1, Kiaa1355-hp, NRT1, Ncaml, mKIAA1355	4q13.2	Candidate	Paternal	TTACTGGCCC	R	BC030141
34	NAP1L5	Nucleosome assembly protein 1-like 5	DRLM	4q22.1	Imprinted	Paternal	TAGCTTTTAG		NM_153757
35	DUX2	Double homeobox 2		4q35.2	Candidate	Paternal	AAGGGGTGGA		NM_012147
36	CDH18	Cadherin 18, type 2	CDH14, CDH24, CDH14L, EY-CADHERIN	5p14.3	Candidate	Paternal	ATCGAAACTG		NM_004934
37	ADAMTS16	ADAM metallopeptidase with thrombospondin type 1 motif, 16	FLJ16731, ADAMTS16s	5p15.32	Candidate	Maternal	TACCCCTGAA	*	AK122980
38	Q8TBP5	Family with sequence similarity 174, member A	FAM174A	5q21.1	Candidate	Paternal	ACCCAGCGGG	*	NM_198507
39	CSF2	Colony-stimulating factor 2 (granulocyte-macrophage)	GMCSF, MGC131935, MGC138897	5q23.3	Candidate	Maternal	GTGGGAGTGG		BC108724
40	BTNL2	Butyrophilin-like 2 (MHC class II associated)	SS2, BTLII, HSBLMHC1	6p21.32	Candidate	Maternal	GAAGGAAAGA		NM_019602
41	FAM50B	Family with sequence similarity 50, member B	X5L, D6S2654E	6p25.2	Imprinted	Paternal	CCTCAGTTTG		BC001261
42	C6orf117	Chromosome 6 open-reading frame 117	MRAP2	6q14.2	Candidate	Paternal	GCAAGCTGTT		NM_138409
43	HYMAI	Hydatidiform mole associated and imprinted (nonprotein coding)	NCRNA00020	6q24.2	Imprinted	Paternal	TATATATTGA		BC059359
44	PLAGL1	Pleiomorphic adenoma gene-like 1	ZAC, LOT1, ZAC1, MGC126275, MGC126276, DKFZp781P1017	6q24–q25	Imprinted	Paternal	ATCATAATGT	*	NM_001080951
45	SLC22A2	Solute carrier family 22 (organic cation transporter), member 2	OCT2, MGC32628	6q26	Imprinted	Maternal	AAAATTATAA		BC030978
46	SLC22A3	Solute carrier family 22 (extraneuronal monoamine transporter), member 3	EMT, EMTH, OCT3	6q26–q27	Imprinted	Maternal	TGCGCTAATC		AF078749
47	BRP44L	Brain protein 44-like	CGI-129, dJ68L15.3	6q27	Candidate	Paternal	CAGTGTATAT		BC000810
48	DDC	Dopa decarboxylase (aromatic L-amino acid decarboxylase)	AADC	7p12.2	Imprinted	Isoform Dependent	TGGCTAAATG		NM_000790
49	GRB10	Growth factor receptor-bound protein 10	RSS, IRBP, MEG1, GRB-IR, Grb-10, KIAA0207	7p12–p11.2	Imprinted	Isoform Dependent	TGCTTTGCTT		NM_001001549
50	GLI3	GLI family zinc finger 3	PHS, ACLS, GCPS, PAPA, PAPB, PAP-A, PAPA1, PPDIV	7p14.1	Candidate	Maternal	TAAATACATT	*	NM_000168
51	EVX1	Even-skipped homeobox 1		7p15.2	Candidate	Paternal	ACGCCCGTGG		NM_001989
52	HOXA5	Homeobox A5	HOX1C, HOX1.3, MGC9376	7p15.2	Candidate	Maternal	AGCCTGTTTA		BC013682
53	HOXA2	Homeobox A2	HOX1K	7p15.2	Candidate	Maternal	CATATTTTTT	*	NM_006735
54	HOXA3	Homeobox A3	HOX1E, MGC10155	7p15.2	Candidate	Maternal	CTCTTCCTCG	R	BC015180
55	HOXA11	Homeobox A11	HOX1I	7p15.2	Candidate	Maternal	GAGATAGCCC		BC040948
56	HOXA4	Homeobox A4	HOX1D	7p15.2	Candidate	Maternal	TGCTAAGAAT		NM_002141
57	TMEM60	Transmembrane protein 60	DC32, MGC74482, C7orf35	7q11.23	Candidate	Paternal	AATCTATCCT		NM_032936
58	PEG10	Paternally expressed 10	Edr, HB-1, Mar2, MEF3L, Mart2, RGAG3, KIAA1051	7q21	Imprinted	Paternal	GAAGTTATAA		NM_001040152
59	MAGI2	Membrane-associated guanylate kinase, WW and PDZ domain containing 2	AIP1, SSCAM, KIAA0705	7q21.11	Candidate	Maternal	TATTAATAGT		BC150277
60	PPP1R9A	Protein phosphatase 1, regulatory (inhibitor) subunit 9A	NRB1, NRBI, FLJ20068, KIAA1222, neurabin-I	7q21.3	Imprinted	Maternal	GAAGAGACAA		NM_017650
61	SGCE	Sarcoglycan, epsilon	ESG, DYT11	7q21–q22	Imprinted	Paternal	TTGGCAGTAT	*	NM_001099400
62	TFPI2	Tissue factor pathway inhibitor 2	PP5, REF1, TFPI-2, FLJ21164	7q22	Imprinted	Maternal	TGCTTTTAAC		NM_006528
63	MEST	Mesoderm-specific transcript homolog (mouse)	PEG1, MGC8703, MGC111102, DKFZp686L18234	7q32	Imprinted	Paternal	CTGAATGTAC		NM_002402
64	COPG2IT1	COPG2 imprinted transcript 1 (nonprotein coding)	CIT1, COPG2AS, FLJ41646, NCRNA00170, DKFZP761N09121	7q32	Imprinted	Paternal	GAGGGATGGC	*	AF038190
65	CPA4	Carboxypeptidase A4	CPA3	7q32	Imprinted	Maternal	TCTGTAAATC	*	BC052289
66	MESTIT1	MEST intronic transcript 1 (nonprotein coding)	PEG1-AS, NCRNA00040	7q32	Imprinted	Paternal	TGTAGTGGTG		NR_004382
67	KLF14	Kruppel-like factor 14	BTEB5	7q32.3	Imprinted	Maternal	TGGACTCTGG		NM_138693
68	SLC4A2	Solute carrier family 4, anion exchanger, member 2 (erythrocyte membrane protein band 3-like 1)	AE2, HKB3, BND3L, NBND3, EPB3L1	7q36.1	Candidate	Maternal	CCCCTCCCTC	*	NM_003040
69	FASTK	Fas-activated serine/threonine kinase	FAST	7q36.1	Candidate	Maternal	GGGGGTGGAT		NM_006712
70	PURG	Purine-rich element binding protein G	PURG-A, PURG-B, MGC119274	8p12	Candidate	Paternal	CTGAACAAAG		NM_001015508
71	DLGAP2	Discs, large (*Drosophila*) homolog-associated protein 2	DAP2, SAPAP2	8p23	Imprinted	Paternal	CCCCAGCCCC	*	NM_004745
72	Q8N9I4	FLJ37098 fis, clone BRACE2019004		8p23.1	Candidate	Paternal	CTAAGCGCAG		AK094417
73	FAM77D	Family with sequence similarity 77, member D	NKAIN3, FLJ39630	8q12.3	Candidate	Paternal	GTGCCCTACC		NM_173688
74	GPT	Glutamic-pyruvate transaminase (alanine aminotransferase)	GPT1, AAT1, ALT1	8q24.3	Candidate	Maternal	CCAAGTTCAC		NM_005309
75	KCNK9	Potassium channel, subfamily K, member 9	KT3.2, TASK3, K2p9.1, TASK-3, MGC138268, MGC138270	8q24.3	Imprinted	Maternal	CCAGGCACTC	*	AK090707
76	LY6D	Lymphocyte antigen 6 complex, locus D	E48	8q24.3	Candidate	Paternal	GAGATAAATG		BC031330
77	APBA1	Amyloid beta (A4) precursor protein-binding, family A, member 1	X11, D9S411E, MINT1, LIN10	9q21.11	Candidate	Paternal	TGTCTCCTTC		NM_001163
78	NM182505	Chromosome 9 open-reading frame 85	C9orf85, MGC61599, RP11-346E17.2	9q21.12	Candidate	Paternal	TAAAAATAAA		NM_182505
79	FAM75D1	Family with sequence similarity 75, member D1	FLJ46321	9q21.32	Candidate	Maternal	CCCCACAGGA		NM_001001670
80	ABCA1	ATP-binding cassette, subfamily A (ABC1), member 1	TGD, ABC1, CERP, ABC-1, HDLDT1, FLJ14958, MGC164864, MGC165011	9q31.1	Imprinted	Unknown	ATGGGGAGAG	*	AK024328
81	LMX1B	LIM homeobox transcription factor 1, beta	NPS1, LMX1.2, MGC138325, MGC142051	9q33.3	Candidate	Maternal	GGAGCCCAGC	*	NM_002316
82	EGFL7	EGF-like-domain, multiple 7	ZNEU1, MGC111117, VE-STATIN, RP11-251M1.2	9q34.3	Candidate	Paternal	GCACAGGCCA		NM_016215
83	PHPT1	Phosphohistidine phosphatase 1	PHP14, CGI-202, HSPC141, bA216L13.10, DKFZp564M173, RP11-216L13.10	9q34.3	Candidate	Maternal	GCCTATGGTC		NM_014172
84	NM144654	Chromosome 9 open-reading frame 116, transcript variant 2	C9orf116, FLJ13945, MGC29761, RP11-426A6.4	9q34.3	Candidate	Paternal	GGAAAGATGC		NM_144654
85	GATA3	GATA binding protein 3	HDR	10p14	Candidate	Paternal	AAGGATGCCA	*	BC003070
86	Q9H6Z8	FLJ21625 fis, clone COL08015		10q23.31	Candidate	Paternal	GCAGCAGCCT		AK025278
87	LDB1	LIM domain binding 1	CLIM2, NLI	10q24.32	Candidate	Maternal	TCCTGACCAC		NM_001113407
88	INPP5F V2	Inositol polyphosphate-5-phosphatase F	SAC2, hSAC2, MSTP007, MSTPO47, FLJ13081, KIAA0966, MGC59773, MGC131851	10q26.11	Imprinted	Paternal	AGATTGAGGC		NR_003252
89	C10orf93	Chromosome 10 open-reading frame 93	bB137A17.3, RP13-137A17.3	10q26.3	Candidate	Maternal	AACAAAATTA		BC044661
90	NKX6-2	NK6 homeobox 2	NK, NKX6B	10q26.3	Candidate	Maternal	ACCGAGAGCC	*	NM_177400
91	PAOX	Polyamine oxidase (exo-N4-amino)	PAO, DKFZp434J245	10q26.3	Candidate	Maternal	GAGACTCTGT		NM_152911
92	C10orf91	Chromosome 10 open-reading frame 91	bA432J24.4, RP11-432J24.4	10q26.3	Candidate	Maternal	GGTTCTCAGC		BC030794
93	VENTX2	VENT-like homeobox-2	NA88A, HPX42B, VENTX2	10q26.3	Candidate	Maternal	TGCTTTTAAA		AF068006
94	WT1-Alt trans	Wilms tumor 1	WT1, GUD, WAGR, WT33, WIT-2	11p13	Imprinted	Paternal	CTGGTATATG		BC032861
95	KCNQ1OT1	KCNQ1 overlapping transcript 1 (nonprotein coding)	LIT1, KvDMR1, KCNQ10T1, KvLQT1-AS, long QT intronic transcript 1	11p15	Imprinted	Paternal	AAATATTTAC		AF086011
96	KCNQ1DN	KCNQ1 downstream neighbor	BWRT, HSA404617	11p15.4	Imprinted	Maternal	GGACCCCAAA		AB039920
97	OSBPL5	Oxysterol binding protein-like 5	ORP5, OBPH1, FLJ42929	11p15.4	Imprinted	Maternal	GGGGATGGAT		NM_001144063
98	PKP3	Plakophilin 3		11p15.5	Candidate	Maternal	AACAGTCAAA		NM_007183
99	Q8N9U2	FLJ36520 fis, clone TRACH2002100		11p15.5	Candidate	Maternal	ACAAGTATTC		AK093839
100	IFITM1	Interferon-induced transmembrane protein 1 (9–27)	IFI17, LEU13, CD225	11p15.5	Candidate	Maternal	ACCATTGGAT		NM_003641
101	PHLDA2	Pleckstrin homology-like domain, family A, member 2	IPL, BRW1C, BWR1C, HLDA2, TSSC3	11p15.5	Imprinted	Maternal	AGCCCGCCGC		NM_003311
102	CDKN1C	Cyclin-dependent kinase inhibitor 1C (p57, Kip2)	BWS, WBS, p57, BWCR, KIP2	11p15.5	Imprinted	Maternal	CCCATCTAGC		NM_000076
103	SLC22A18	Solute carrier family 22, member 18	HET, ITM, BWR1A, IMPT1, TSSC5, ORCTL2, BWSCR1A, SLC22A1L, p45-BWR1A, DKFZp667A184	11p15.5	Imprinted	Maternal	CTGGGCCTCT	*	NM_002555
104	IGF2/INS	Insulin/insulin-like growth factor 2 (somatomedin A)	INSIGF, pp9974, C11orf43, FLJ22066, FLJ44734/ILPR, IRDN	11p15.5	Imprinted	Paternal	CTTGGGTTTT		BC011786
105	IGF2AS	Insulin-like growth factor 2 antisense	PEG8, MGC168198	11p15.5	Imprinted	Paternal	GAGGGCCGTT		AB030733
106	H19	H19, imprinted maternally expressed transcript (nonprotein coding)	ASM, BWS, ASM1, MGC4485, PRO2605, D11S813E	11p15.5	Imprinted	Maternal	GCCACCCCCT	*	BC007513
107	KCNQ1	Potassium voltage-gated channel, KQT-like subfamily, member 1	LQT, RWS, WRS, LQT1, SQT2, ATFB1, ATFB3, JLNS1, KCNA8, KCNA9, Kv1.9, Kv7.1, KVLQT1, FLJ26167	11p15.5	Imprinted	Maternal	GGCAGGAGAC		BC017074
108	B4GALNT4	Beta-1,4-N-acetyl-galactosaminyl transferase 4	FLJ25045	11p15.5	Candidate	Maternal	TGGAGCGTCC		NM_178537
109	RAB1B	RAB1B, member RAS oncogene family		11q13.2	Candidate	Maternal	TCAGGCATTT		BC071169
110	KBTBD3	Kelch repeat and BTB (POZ) domain containing 3	BKLHD3, FLJ30685	11q22.3	Candidate	Paternal	AAACTACAAA		AK092993
111	NTRI	Neurotrimin	NTM, HNT, IGLON2, MGC60329	11q25	Candidate	Paternal	TCCCTCTTCA	R	NM_016522
112	ABCC9	ATP-binding cassette, subfamily C (CFTR/MRP), member 9	SUR2, ABC37, CMD1O, FLJ36852	12p12.1	Candidate	Maternal	TGTCTTTAAA	*	BX537513
113	RBP5	Retinol binding protein 5, cellular	CRBP3, CRBPIII, CRBP-III	12p13.31	Imprinted	Maternal	CTTCCTGTTA	*	AK096947
114	HOXC4	Homeobox C4	HOX3E, CP19	12q13.13	Candidate	Maternal	GTACCTGCTG		NM_153633
115	HOXC9	Homeobox C9	HOX3B	12q13.13	Candidate	Maternal	TACGGCTCGC		BC032769
116	SLC26A10	Solute carrier family 26, member 10		12q13.3	Candidate	Maternal	ACCCTTGAAC		NM_133489
117	CDK4	Cyclin-dependent kinase 4	PSK-J3, CMM3	12q14.1	Candidate	Maternal	GAAGGAAGAA	*	BC005864
118	Q96AV8	E2F transcription factor 7	E2F7, FLJ12981	12q21.2	Candidate	Maternal	TAAACTGATT		BC016658
119	Q9HCM7	Fibrosin-1-like protein	FBRSL1, AUTS2L, KIAA1545, XTP9	12q24.33	Candidate	Maternal	TCAATCAGTG		NM_001142641
120	Q8N7V5	Proline-rich 20A	PRR20A, FLJ40296	13q21.1	Candidate	Maternal	ACTCACTGGA	*	NM_198441
121	FAM70B	Family with sequence similarity 70, member B		13q34	Candidate	Maternal	GTGCCTCTGT		NM_182614
122	FOXG1C	Forkhead box G1	HFK3	14q12	Candidate	Paternal	GAACTATATG		BC050072
123	PLEKHC1	Fermitin family (*Drosophila*) homolog 2	FERMT2, MIG2, UNC112, KIND2	14q22.1	Candidate	Paternal	GTTCAAAGAC		NM_001134999
124	DLK1	Delta-like 1 homolog (*Drosophila*)	DLK, FA1, ZOG, pG2, PREF1, Pref-1	14q32	Imprinted	Paternal	ATACAGAATA	*	BC013197
125	MEG3	Maternally expressed 3 (nonprotein coding)	GTL2, FP504, prebp1, PRO0518, PRO2160, FLJ31163, FLJ42589	14q32	Imprinted	Maternal	TGGGAAGTGG		AB032607
126	RTL1	Retrotransposon-like 1	PEG11	14q32.31	Candidate	Maternal	ACGGCCTGCA		NM_001134888
127	ATP10A	ATPase, class V, type 10A	ATPVA, ATPVC, ATP10C, KIAA0566	15q11.2	Imprinted	Maternal	GCCCCCAGAG		BC052251
128	PWCR1	Prader-Willi syndrome chromosome region 1	PET1, noncoding RNA in the Prader-Willi critical region	15q11.2	Imprinted	Paternal	TTGGTGAGGG		AF241255
129	NDN	Necdin homolog (mouse)	HsT16328	15q11.2–q12	Imprinted	Paternal	ACCTTGCTGG		BC008750
130	SNURF/ SNRPN	SNRPN upstream reading frame/small nuclear ribonucleoprotein polypeptide N	SMN, PWCR, SM-D, RT-LI, HCERN3, SNRNP-N, FLJ33569, FLJ36996, FLJ39265, MGC29886, SNURF-SNRPN, DKFZp762N022, DKFZp686C0927, DKFZp761I1912, DKFZp686M12165	15q11.2–q12	Imprinted	Paternal	CCGCCTCCGG		BC000611
131	MAGEL2	MAGE-like 2	nM15, NDNL1	15q11–q12	Imprinted	Paternal	TAGCATTGTA		BC035839
132	MKRN3	Makorin ring finger protein 3	D15S9, RNF63, ZFP127, ZNF127, MGC88288	15q11–q13	Imprinted	Paternal	AAATAATTTA		NM_005664
133	UBE3A	Ubiquitin protein ligase E3A	AS, ANCR, E6-AP, HPVE6A, EPVE6AP, FLJ26981	15q11–q13	Imprinted	Maternal	CTGTAAAACA		BC002582
134	Q9P168	PRO2369		15q13.1	Candidate	Paternal	AGAACTCCAC		AF119879
135	SOX8	SRY (sex-determining region Y)-box 8		16p13.3	Candidate	Paternal	CAGCGTCTCC		BC031797
136	SALL1	Sal-like 1 (*Drosophila*)	HSAL1	16q12.1	Candidate	Maternal	ACATTTCTAG	R	BC113881
137	C16orf57	Chromosome 16 open-reading frame 57		16q13	Candidate	Maternal	GGATTTTAAT		BC004415
138	ACD	Adrenocortical dysplasia homolog (mouse)	PTOP, PIP1, TINT1, TPP1	16q22.1	Candidate	Maternal	CGGCAAAAAA		BC016904
139	FOXF1	Forkhead box F1	FKHL5, FREAC1, ACDMPV	16q24.1	Candidate	Maternal	TTCCTCCTCT	*	BC089442
140	ANKRD11	Ankyrin repeat domain 11	T13, LZ16, ANCO-1	16q24.3	Imprinted	Maternal	AAAGCTGACA		BC058001
141	Q8N206	FLJ36443 fis, clone THYMU2012891	FLJ36443 fis	16q24.3	Candidate	Maternal	ACATTCAGAA		AK093762
142	TMEM88	Transmembrane protein 88	FLJ20025	17p13.1	Candidate	Maternal	CTGGGCTTCG		NM_203411
143	PYY2	Peptide YY, 2 (seminal plasmin)		17q11.2	Candidate	Paternal	TTCACTCCCG		AF222904
144	HOXB3	Homeobox B3	HOX2G	17q21.32	Candidate	Maternal	AACTCAGCTC		NM_002146
145	HOXB2	Homeobox B2	HOX2H	17q21.32	Candidate	Maternal	AAGCACAAGC		NM_002145
146	Q8N8L1	FLJ39287 fis, clone OCBBF2011897	LOC100131170	17q25.3	Candidate	Paternal	GGGTCTGAGG		AK096606
147	FAM59A	Family with sequence similarity 59, member A	GAREM, Gm944, C18orf11	18q12.1	Candidate	Paternal	TGCAGAGAAA		NM_022751
148	BRUNOL4	Bruno-like 4	CELF4	18q12.2	Candidate	Maternal	GCTGTTCTTG		NM_001025087
149	TCEB3C	Transcription elongation factor B polypeptide 3C (elongin A3)	HsT829, TCEB3L2, elongin A3	18q21.1	Imprinted	Maternal	ACCTCCCAGG	*	NM_145653
150	Q8NE65	Zinc finger protein 738	ZNF738	19p13.11	Candidate	Paternal	TTGGTCAGGC	R	BC034499
151	Q8NB05	FLJ34424 fis, clone HHDPC2008279		19p13.2	Candidate	Paternal	TGCTCGGGAA		AK091743
152	PPAP2C	Phosphatidic acid phosphatase type 2C	PAP2C, LPP2	19p13.3	Candidate	Maternal	GTGTTCTTGG		NM_003712
153	TSH3	Teashirt zinc finger homeobox 3	TSHZ3, ZNF537, FLJ54422, KIAA1474	19q12	Candidate	Paternal	TTCTTATTTT	*	AK291466
154	CHST8	Carbohydrate (N-acetylgalactosamine 4-0) sulfotransferase 8	GalNAc4ST1, GalNAc4ST	19q13.11	Candidate	Maternal	GTTTCCAGAG	*	NM_001127895
155	ZNF225	Zinc finger protein 225	MGC119735	19q13.31	Candidate	Paternal	TGGTATGTAT		NM_013362
156	ZNF229	Zinc finger protein 229	FLJ34222	19q13.31	Candidate	Maternal	TTGTAACCTC		NM_014518
157	ZNF264	Zinc finger protein 264	ZFP264	19q13.4	Imprinted	Maternal	GCTTCAGTGG		NM_003417
158	ZIM2/PEG3	ZIM2 zinc finger, imprinted 2/Paternally expressed 3	ZNF656/PW1, ZSCAN24, KIAA0287, DKFZp781A095	19q13.4	Imprinted	Paternal	TTTTCACCAT		BC037330
159	LILRB4	Leukocyte immunoglobulin-like receptor, subfamily B (with TM and ITIM domains), member 4	LIR5, ILT3, HM18, CD85K	19q13.42	Candidate	Maternal	GGAAAATGGG	*	NM_001081438
160	ZNF550	Zinc finger protein 550		19q13.43	Candidate	Maternal	AGAAATGTAC	*	AK122867
161	CHMP2A	Chromatin-modifying protein 2A	VPS2A, VPS2, BC2	19q13.43	Candidate	Maternal	GGTGATGAGG	*	NM_014453
162	ZNF42	Zinc finger protein 42	MZF1, MZF1B, ZFP98, ZSCAN6	19q13.43	Candidate	Maternal	GTCAGAACAC	*	NM_003422
163	ISM1	Isthmin 1 homolog (zebrafish)	C20orf82	20p12.1	Candidate	Paternal	AATATTATCA		NM_080826
164	NNAT	Neuronatin	PEG5, MGC1439	20q11.2–q12	Imprinted	Paternal	CAGTTGTGGT		NM_005386
165	BLCAP	Bladder cancer-associated protein	BC10	20q11.2–q12	Imprinted	Isoform Dependent	CCTGTCCTTT		NM_006698
166	L3MBTL	L(3)mbt-like (*Drosophila*)	L3MBTL1, FLJ41181, KIAA0681, H-L(3)MBT, dJ138B7.3, DKFZp586P1522	20q13.12	Imprinted	Paternal	TGTGTATGTG	*	AB014581
167	GNAS	GNAS complex locus	AHO, GSA, GSP, POH, GPSA, NESP, GNAS1, PHP1A, PHP1B, C20orf45, MGC33735, dJ309F20.1.1, dJ806M20.3.3	20q13.3	Imprinted	Isoform Dependent	ATTAACAAAG		NM_000516
168	GNASAS	GNAS antisense RNA 1 (nonprotein coding)	SANG, NESPAS, GNAS1AS, NCRNA00075	20q13.32	Imprinted	Paternal	TCCATTAGAA		AJ251759
169	COL9A3	Collagen, type IX, alpha 3	IDD, MED, EDM3, FLJ90759, DJ885L7.4.1	20q13.33	Candidate	Maternal	AAGGAGCGGG	*	BC011705
170	C20orf20	Chromosome 20 open-reading frame 20	Eaf7, MRGBP, URCC4, MRG15BP, FLJ10914	20q13.33	Candidate	Maternal	ACCTCACTCT		BC009889
171	SIM2	Single-minded homolog 2 (*Drosophila*)	SIM, bHLHe15, MGC119447	21q22.13	Candidate	Paternal	AAGGAAGATT	*	NM_005069
172	DGCR6	DiGeorge syndrome critical region gene 6		22q11.21	Candidate	Paternal	CAGAAGAGGC	*	NM_005675
173	FLJ20464	Hypothetical protein FLJ20464		22q12.2	Candidate	Paternal	CGTGAAATTC		CR456348

SAGE tags annotated for *NlaIII* anchoring enzyme.

^a^Entries are sorted according to the established gene location.

^b∗^: tag maps to other gene(s) according to CGAP (Cancer Genome Anatomy Project, NCI, NIH) SAGE Anatomic Viewer.

^c#^: highly repetitive tag according to CGAP SAGE Anatomic Viewer.

^d^R: unreliable/internal tag suggested by CGAP SAGE Anatomic Viewer is replaced with reliable 3′ end tag.

**Table 2:** Summary of SAGE catalogs analyzed.

Clusters	Number of SAGE catalogs^a^	Number of SAGE tags^b^
C (cancer tissue)	185	13,165,432
N (normal tissue and cells)	166	12,953,131
IV (cells cultured *in vitro*)	112	8,009,673
D (nontumorous disease tissue and cells)	29	1,840,291

Total	492	35,968,527

All SAGE catalogs screened belong to GPL4 Gene Expression Omnibus database (GEO, NCBI) platform (*Homo sapiens*; *NlaIII*-anchoring enzyme).

^a^SAGE catalogs selected for analysis (see Supplementary Table 1 available online at doi:10.1155/2012/793506).

^b^Number of tags subjected for analysis (with (A)_10_ tags excluded).

**Table 3:** The SAGE libraries with 10% most and 10% least cumulative and average expression of established and candidate imprinted genes subsets.

ID^a^	Primary ID^b^	SAGE library	Sample	Cluster^c^	Sum^d^	Average^e^	Max^f^
Top 10% libraries							

76	301	GSM125353	Bronchial brushings, former smoker	N	43,563.92	251.81	40,054.67
4	6	GSM574	Central retina (macula)	N	33,159.24	191.67	26,692.01
142	427	GSM383793	Mammary gland, ductal carcinoma *in situ *	C	29,781.50	172.15	25,275.20
29	104	GSM1730	Breast, ductal carcinoma *in situ *	C	29,575.98	170.96	25,125.63
75	300	GSM125352	Bronchial brushings, former smoker	N	29,184.64	168.70	24,955.09
145	430	GSM383797	Mammary gland, ductal carcinoma	C	27,222.30	157.35	22,422.27
99	346	GSM194651	Oral biopsy	N	27,066.16	156.45	21,312.29
90	273	GSM112808	Neuroblastoma, primary tumor, stage 4S	C	25,944.14	149.97	17,365.83
55	155	GSM14753	Breast carcinoma metastasis to lung	C	23,941.22	138.39	20,247.08
143	428	GSM383794	Mammary gland, ductal carcinoma *in situ *	C	23,570.83	136.25	18,977.90
30	105	GSM1731	Breast, ductal carcinoma *in situ *	C	23,346.84	134.95	18,746.14
167	507	GSM383893	Gallbladder tubular adenocarcinoma	C	23,262.07	134.46	20,154.48
27	561	GSM384016	Vascular endothelium, hemangioma, benign hyperplasia	D	22,688.19	131.15	12,514.88
91	274	GSM112809	Neuroblastoma, primary tumor, stage 4S	C	22,633.99	130.83	11,595.94
28	101	GSM1516	Hemangioma tumor	C	22,622.41	130.77	12,500.82
100	347	GSM194652	Oral biopsy	N	21,279.52	123.00	11,932.33
146	440	GSM383807	Mammary gland, ductal carcinoma *in situ *	C	20,570.15	118.90	16,071.37
62	433	GSM383800	Breast carcinoma cell line	IV	20,467.40	118.31	12,710.80
140	425	GSM383790	Mammary gland, ductal carcinoma	C	20,417.00	118.02	15,536.62
144	537	GSM383946	Whole body, fetal	N	20,274.33	117.19	9,698.99
149	443	GSM383812	Mammary gland, ductal carcinoma	C	20,036.49	115.82	13,377.93
5	21	GSM688	Breast, ductal carcinoma *in situ *	C	19,968.16	115.42	15,538.48
118	416	GSM383775	Cortex, pooled sample	N	19,926.52	115.18	13,701.49
54	263	GSM85616	Bronchial epithelium	N	19,665.60	113.67	15,918.74
53	262	GSM85611	Bronchial epithelium	N	19,653.98	113.61	15,655.95
17	340	GSM194386	Metaplastic bronchial epithelium	D	19,376.31	112.00	14,577.13
81	306	GSM125358	Bronchial brushings, never smoker	N	19,369.05	111.96	14,172.30
137	509	GSM383895	Gallbladder	N	19,320.00	111.68	15,346.14
66	437	GSM383804	Breast carcinoma cell line	IV	19,247.28	111.26	9,389.55
31	106	GSM1733	Mammary gland, ductal invasive *in situ* carcinoma	C	19,126.03	110.56	14,594.68
92	276	GSM112812	Neuroblastoma, primary tumor, stage 4	C	18,914.78	109.33	12,349.04
3	5	GSM573	Peripheral retina	N	18,817.72	108.77	12,293.99
2	4	GSM572	Peripheral retina	N	18,781.47	108.56	8,727.76
96	331	GSM194377	Nonsmall cell lung cancer: squamous cell carcinoma *in situ *	C	18,122.08	104.75	14,601.68
71	181	GSM14781	Brain desmoplastic medulloblastoma	C	18,106.48	104.66	11,244.70
68	439	GSM383806	Breast carcinoma cell line	IV	17,991.89	104.00	8,886.81
66	291	GSM125343	Bronchial brushings, former smoker	N	17,662.13	102.09	12,756.35
60	285	GSM125337	Bronchial brushings, current smoker	N	17,598.56	101.73	12,699.28
121	387	GSM383710	Ependymoma	C	17,524.27	101.30	10,222.49
51	260	GSM82458	Hippocampus	N	17,289.52	99.94	8,268.90
72	297	GSM125349	Bronchial brushings, former smoker	N	17,225.16	99.57	13,193.26
109	361	GSM296391	Lung biopsy	N	17,223.67	99.56	13,839.55
62	287	GSM125339	Bronchial brushings, current smoker	N	16,871.13	97.52	10,737.09
98	333	GSM194379	Nonsmall cell lung cancer: squamous cell carcinoma* in situ *	C	16,572.81	95.80	12,713.33
74	299	GSM125351	Bronchial brushings, former smoker	N	16,456.57	95.12	11,137.34
70	295	GSM125347	Bronchial brushings, former smoker	N	16,306.85	94.26	11,835.62
7	216	GSM37212	Adrenal cortex affected by primary pigmented nodular adrenocortical disease	D	16,221.46	93.77	4,205.56
59	284	GSM125336	Bronchial brushings, current smoker	N	16,090.33	93.01	11,242.21
94	329	GSM194375	Nonsmall cell lung cancer: squamous cell carcinoma *in situ *	C	15,715.04	90.84	11,679.51

Bottom 10% libraries							

83	488	GSM383868	Colon carcinoma, cell line	IV	4,179.31	24.16	422.91
38	180	GSM14780	Gastric epithelial tissue from the antrum	N	4,169.95	24.10	1,615.39
84	204	GSM14807	Lung, poorly differentiated adenocarcinoma with lymphoplasmacytic infiltration	C	4,146.18	23.97	460.69
181	553	GSM383998	Gastroesophageal junction adenocarcinoma	C	4,141.08	23.94	1,351.60
1	7	GSM668	Kidney, embryonic cell line 293, uninduced cells	IV	4,119.01	23.81	920.45
4	120	GSM3244	AIDS-KS lesion	D	4,107.85	23.74	1,245.47
25	522	GSM383914	Lung, tumor associated (focal fibrosis and chronic inflammation)	D	4,103.62	23.72	606.45
30	121	GSM3245	CD4+ T cells	N	4,088.74	23.63	978.17
44	137	GSM14734	Medulloblastoma, cerebellum	C	4,081.98	23.60	804.90
56	162	GSM14760	Stomach, poorly differentiated carcinoma	C	4,081.16	23.59	1,340.95
54	153	GSM14751	Skin, melanoma	C	4,072.22	23.54	2,497.12
80	485	GSM383865	Colon carcinoma, cell line	IV	4,048.02	23.40	404.80
130	404	GSM383753	Medulloblastoma	C	3,987.16	23.05	1,069.73
82	487	GSM383867	Colon carcinoma, cell line	IV	3,961.32	22.90	506.24
12	71	GSM747	Colon, cancer cell line	IV	3,945.42	22.81	673.61
166	506	GSM383892	Gallbladder adenocarcinoma	C	3,909.95	22.60	651.66
115	372	GSM311354	CD15+ myeloid progenitor cells	N	3,859.27	22.31	1,108.11
52	261	GSM82459	Spermatozoa	N	3,814.90	22.05	1,288.15
50	259	GSM82243	Spermatozoa, pooled sample	N	3,811.16	22.03	1,429.18
94	328	GSM180670	Lymphocytes from children 1–4 years old (pooled samples)	N	3,794.27	21.93	843.17
81	486	GSM383866	Colon carcinoma, cell line	IV	3,746.12	21.65	378.40
39	183	GSM14784	Bone marrow	N	3,740.58	21.62	962.65
88	318	GSM136195	Cord blood-derived activated Th1 cells	N	3,668.81	21.21	777.29
20	423	GSM383787	Breast stroma, ductal carcinoma *in situ* associated	D	3,663.78	21.18	334.67
38	234	GSM66698	HL-60 cells	IV	3,661.81	21.17	920.25
168	508	GSM383894	Gallbladder tubular adenocarcinoma	C	3,643.40	21.06	624.22
28	217	GSM37337	Primary bronchial epithelial cells	IV	3,604.30	20.83	804.84
177	546	GSM383970	Retinoblastoma	C	3,561.82	20.59	384.45
71	475	GSM383852	Cartilage chondrosarcoma cell line	IV	3,502.94	20.25	515.14
38	127	GSM7800	Primary gastric cancer, poorly differentiated (scirrhous type)	C	3,413.27	19.73	636.37
130	466	GSM383840	Mammary myoepithelium, CD10+ cells	N	3,364.85	19.45	291.33
39	235	GSM66712	HL-60 cells exposed to 2.45 GHz radiofrequency for 2 h	IV	3,293.77	19.04	982.36
97	344	GSM194649	Oral brushing	N	3,290.72	19.02	996.85
173	523	GSM383915	Lymph node, B-cell lymphoma	C	3,166.97	18.31	555.61
140	514	GSM383902	Leukocytes	N	2,991.69	17.29	439.34
65	436	GSM383803	Breast carcinoma cell line	IV	2,988.12	17.27	597.62
63	434	GSM383801	Breast carcinoma cell line	IV	2,977.71	17.21	297.77
21	84	GSM784	Gastric epithelial tissues	N	2,970.06	17.17	673.21
151	554	GSM384002	Stomach	N	2,813.83	16.26	654.38
170	515	GSM383903	Liver cholangiocarcinoma metastasis	C	2,736.32	15.82	1,575.46
29	578	GSM389908	Total blood after EPO treatment, pooled sample	D	2,684.90	15.52	747.69
68	175	GSM14775	Skin, primary malignant melanoma	C	2,612.99	15.10	653.25
40	236	GSM66714	HL-60 cells exposed to 2.45 GHz radiofrequency for 6 h	IV	1,879.82	10.87	639.52
143	533	GSM383937	Pancreas	N	1,813.11	10.48	278.94
83	308	GSM135389	Skeletal muscle, 5 days training young men	N	1,673.61	9.67	435.14
86	311	GSM135392	Skeletal muscle, detraining young men	N	1,670.43	9.66	510.41
165	576	GSM389906	Total blood, pooled sample	N	1,511.12	8.73	436.55
82	307	GSM135388	Skeletal muscle, pretraining young men	N	1,091.72	6.31	327.52
28	577	GSM389907	Total blood during EPO treatment, pooled sample	D	811.75	4.69	162.35

Indexes (GSM numbers) represent GEO database accession numbers for SAGE libraries (one accession number selected for redundant entries).

^a^ID: listing within each cluster (see Supplementary Table 2).

^b^Primary ID: listing within a full dataset (see Supplementary Table 1).

^c^Clusters: C: cancer tissue; N: normal tissue and cells; IV: cells cultured *in vitro*; D: nontumorous disease tissue and cells.

^d^Sum: cumulative (total) tag per million (tpm) value for SAGE tags matching established and candidate imprinted genes within the SAGE library.

^e^Average: tpm value for SAGE tags matching established and candidate imprinted genes within the SAGE library.

^f^Max: maximum tpm value for SAGE tags matching established and candidate imprinted genes within the SAGE library.

Particular sum: average and maximum values could be recalculated to the fraction of the total gene expression by dividing tpm value to 1,000,000.

Entries are sorted according to the cumulative (total) tpm value.
