# Supplementary material for: A Prevalence of Imprinted Genes within the Total Transcriptomes of Human Tissues and Cells
Source: Mol Biol Int. 2012 Sep 11;2012:793506. doi: 10.1155/2012/793506 (PMC3446743; doi:10.1155/2012/793506)
Supplement: Supplementary file 1 — Supplementary Material provides key properties of established and candidate imprinted gene subset within the SAGE datasets. [file 793506.f1.pdf]

Supplemental Table 1. Particulars of SAGE libraries used in the study.

Primary ID	SAGE Library	Sample	Selection [a]	Tags [b]	Notes [c]	Cluster [d]
1	GSM1	Foreskin fibroblasts	x		9,742 tags	
2	GSM2	HCMV-infected foreskin fibroblasts	-			
3	GSM571	Retinal pigment epithelium (RPE)	+	53,655		N
4	GSM572	Peripheral retina	+	59,580		N
5	GSM573	Peripheral retina	+	104,848		N
6	GSM574	Central retina (macula)	+	102,053		N
7	GSM668	Kidney, embryonic cell line 293, uninduced cells	+	43,457		IV
8	GSM669	Kidney, induced embryonic cell line 293, expressing β-catenin	-			
9	GSM670	Breast tumor	+	40,164		C
10	GSM671	Metastasis from breast tumor	+	45,611		C
11	GSM674	Cell line induced with synthetic androgen (R1881)	-			
12	GSM675	Ovary	+	22,259		N
13	GSM676	Brain, greater than 95% white matter	+	94,652		N
14	GSM677	Breast, luminar mammary epithelium	+	37,565		N
15	GSM678	Pancreas	+	37,894		N
16	GSM680	Prostate carcinoma cell line	+	41,450		IV
17	GSM683	Prostate carcinoma cell line	+	62,493		IV
18	GSM685	Prostate	+	66,384		N

Primary ID	SAGE Library	Sample	Selection [a]	Tags [b]	Notes [c]	Cluster [d]
19	GSM686	Prostate tumor	+	68,540		C
20	GSM687	Breast, ductal carcinoma in situ	+	41,274		C
21	GSM688	Breast, ductal carcinoma in situ	+	28,896		C
22	GSM689	Primary tumor, well-differentiated oligodendroglioma	+	28,112		C
23	GSM690	Brain, medulloblastoma grade IV	+	38,931		C
24	GSM691	Mammary gland, ductal epithelium	x		7,139 tags	
25	GSM692	Mammary gland, ductal epithelium	x		12,123 tags	
26	GSM693	Medulloblastoma tumor	x		19,504 tags	
27	GSM694	Mammary gland, ductal carcinoma	x		5,608 tags	
28	GSM695	Brain, greater than 95% white matter	+	58,770		N
29	GSM696	Brain, Duke glioblastoma multiforme primary tumor	+	69,779		C
30	GSM697	Brain, anaplastic astrocytoma, primary tumor	+	52,432		C
31	GSM698	Pilocytic astrocytoma, primary tumor	+	76,972		C
32	GSM699	Brain, astrocytoma	+	28,150		C
33	GSM700	Brain, glioblastoma multiforme	+	71,840		C
34	GSM701	Brain, glioblastoma multiforme	+	60,477		C
35	GSM703	Brain, Duke glioblastoma multiforme cell line	+	57,549		IV
36	GSM704	Brain, glioblastoma multiforme cell line, stably transfected with epidermal growth factor receptor vIII mutant	-			

Primary ID	SAGE Library	Sample	Selection [a]	Tags [b]	Notes [c]	Cluster [d]
37	GSM705	Brain, glioblastoma multiforme cell line, stably transfected with the β-galactosidase gene	-			
38	GSM706	Microvascular endothelial cells (HMVEC)	+	52,447		N
39	GSM707	Microvascular endothelial cells (HMVEC) treated with VEGF	-			
40	GSM708	Kidney	+	41,652		N
41	GSM710	Brain, c-myc negative medulloblastoma cell line mhh-1	+	48,370		IV
42	GSM711	Skin, post-crisis survival fibroblast cell-line	+	22,317		IV
43	GSM712	Skin, large T antigen transformed fibroblasts clones, precrisis	x		8,783 tags	
44	GSM713	Brain, thalamus	+	48,502		N
45	GSM714	Ovarian clear cell carcinoma cell line ES-2, poorly differentiated	+	31,484		IV
46	GSM715	Brain, primary tumor, well-differentiated, fibrillary astrocytoma	x		17,558 tags	
47	GSM717	Colon, cell line derived from colorectal carcinoma	+	60,310		IV
48	GSM718	Mammary gland epithelium	x		1,423 tags	
49	GSM719	Ovary, surface epithelium	+	48,421		N
50	GSM721	Pancreas, duct epithelial cells	+	32,224		N
51	GSM722	Ovarian surface epithelium cell line	+	48,415		IV
52	GSM723	Prostate, advanced tumor	+	23,009		C
53	GSM725	Mammary gland, adenocarcinoma	x		18,994 tags	

Primary ID	SAGE Library	Sample	Selection [a]	Tags [b]	Notes [c]	Cluster [d]
54	GSM726	Ovary cystadenoma	+	56,950		C
55	GSM727	Abdominal peritoneum, primary mesothelioma (pure epithelial)	+	35,076		C
56	GSM729	Colonic epithelium	+	49,554		N
57	GSM730	Brain, astrocyte cells	+	52,140		IV
58	GSM731	Ovarian carcinoma	x		17,441 tags	
59	GSM732	Ovary, carcinoma cell line	+	38,895		IV
60	GSM733	Ovary, carcinoma cell line	x		2,871 tags	
61	GSM734	Ovary, pooled cancer cell lines	x		10,884 tags	
62	GSM735	Primary ovarian tumor, serous adenocarcinoma	+	42,296		C
63	GSM736	Primary ovarian tumor, serous adenocarcinoma	+	54,934		C
64	GSM737	Primary ovarian tumor, serous adenocarcinoma	+	33,551		C
65	GSM738	Peritoneum, sheet (>90% mesothelial cells)	+	53,976		N
66	GSM740	Prostate, adenocarcinoma	+	65,252		C
67	GSM741	Breast, cancer cell line	x		9,372 tags	
68	GSM743	Pancreas tumor	+	33,924		C
69	GSM744	Pancreas tumor	+	35,716		C
70	GSM746	Prostate, carcinoma	x		9,232 tags	
71	GSM747	Colon, cancer cell line	+	51,959		IV
72	GSM748	Mammary gland, adenocarcinoma, malignant effusion	x		8,094 tags	

Primary ID	SAGE Library	Sample	Selection [a]	Tags [b]	Notes [c]	Cluster [d]
73	GSM751	Breast carcinoma MCF7 cells	x		5,890 tags	
74	GSM754	Prostate, cell line	x		11,363 tags	
75	GSM756	Colon adenocarcinoma	+	49,017		C
76	GSM757	Stomach, primary gastric cancer, gastroesophageal junction adenocarcinoma	+	65,855		C
77	GSM758	Stomach, primary gastric cancer tissue propagated through nude mice (xenograft)	-			
78	GSM759	Breast, cancer cell line	x		18,472 tags	
79	GSM760	Mammary gland, ductal epithelium	+	49,208		N
80	GSM764	Prostate	x		13,293 tags	
81	GSM765	Brain, glioblastoma multiforme primary tumor	+	61,795		C
82	GSM780	Breast tissue (gestational hyperplasia)	+	63,206		N
83	GSM781	Breast, mammary myoepithelial cells	+	58,432		N
84	GSM784	Gastric epithelial tissues	+	25,252		N
85	GSM786	Frontal cortex	+	77,570		N
86	GSM792	Brain, ependymoblastoma, primary tumor	+	34,486		C
87	GSM819	Muscle, old	+	53,853		N
88	GSM824	Muscle, young	+	53,875		N
89	GSM1121	Cultured epidermal keratinocytes	x		12,799 tags	

Primary ID	SAGE Library	Sample	Selection [a]	Tags [b]	Notes [c]	Cluster [d]
90	GSM1122	Cultured epidermal keratinocytes, stimulated with TNF-α	-			
91	GSM1123	Epidermis	x		15,150 tags	
92	GSM1124	Epidermis	x		11,556 tags	
93	GSM1125	Affected epidermis of kidney transplant recipient suffering from actinic keratosis (AK)	x		10,074 tags	
94	GSM1128	Breast cancer cell line	x		1 tag (-)	
95	GSM1129	Breast cancer cell line, estrogen-treated	-			
96	GSM1130	Breast cancer cell line, tamoxifen-treated	-			
97	GSM1498	Brain, glioblastoma multiforme (glioma)	+	62,629		C
98	GSM1499	Heart	+	84,153		N
99	GSM1514	PC3 cells, JNK2AS treatment	-		*	
100	GSM1515	PC3 cells	+	38,819	*	IV
101	GSM1516	Hemangioma tumor	+	76,075		C
102	GSM1652	Corneal endothelium	x		9,536 tags	
103	GSM1653	Fuchs' corneal endothelium	x		9,874 tags	
104	GSM1730	Breast, ductal carcinoma in situ	+	60,894		C
105	GSM1731	Breast, ductal carcinoma in situ	+	43,689		C
106	GSM1733	Mammary gland, ductal invasive in situ carcinoma	+	69,957		C

Primary ID	SAGE Library	Sample	Selection [a]	Tags [b]	Notes [c]	Cluster [d]
107	GSM1734	Pooled organs and tissues include: B-lymphocyte (plasmacytoma, mycloma), mammary gland (adenocarcinoma), liver (hepatoblastoma), cervix (adenocarcinoma), testis (embryonal carcinoma), brain (glioblastoma), melanoma, liposarcoma, macrophage (histiocytic lymphoma, histocyte), and T-lymphoblast (lymphoblastic leukemia) // Universal reference	+	51,711		N
108	GSM2384	Brain, ependymoma	+	52,871		C
109	GSM2385	Primary gastric cancer	+	63,882		C
110	GSM2386	Spinal cord	+	55,199		N
111	GSM2408	Brain, ependymoma	+	52,579		C
112	GSM2443	Brain, astrocytoma	+	80,165		C
113	GSM2451	Brain, astrocytoma	+	38,603		C
114	GSM2455	Uterine cervix	+	30,344		N
115	GSM2578	Brain, astrocytoma	+	69,447		C
116	GSM3240	Nodular skin lesion of an AIDS-KS patient	+	45,907		D
117	GSM3241	Nodular skin lesion of an AIDS-KS patient	+	47,311		D
118	GSM3242	Skin	+	37,247		N
119	GSM3243	AIDS-KS lesion	+	45,973		D

Primary ID	SAGE Library	Sample	Selection [a]	Tags [b]	Notes [c]	Cluster [d]
120	GSM3244	AIDS-KS lesion	+	45,766		D
121	GSM3245	CD4+ T-cells	+	51,116		N
122	GSM3838	Pancreatic exocrine cells	x		17,768 tags	
123	GSM3839	Pancreatic islets	x		18,765 tags	
124	GSM3840	Pancreatic islets	x		12,252 tags	
125	GSM4650	Thyroid	x		10,982 tags	
126	GSM7498	Type 2 Gaucher patient cerebral cortex gray matter	-			
127	GSM7800	Primary gastric cancer, poorly differentiated (scirrhous type)	+	34,571		C
128	GSM8505	Primary gastric cancer	+	31,974		C
129	GSM8867	Primary gastric cancer	+	43,633		C
130	GSM9103	Primary gastric cancer, poorly differentiated (diffuse-adherent type),	x		11,545 tags	
131	GSM9104	Lymph node metastasis from gastric cancer	x		15,312 tags	
132	GSM9220	Undifferentiated HES3 40P embryonic stem cells	+	67,632		IV
133	GSM9221	Undifferentiated HES4 40P embryonic stem cells	+	76,983		IV
134	GSM14731	Medulloblastoma, cerebellum	+	52,200		C
135	GSM14732	Medulloblastoma, cerebellum	+	48,287		C
136	GSM14733	Medulloblastoma, cerebellum	+	42,903		C
137	GSM14734	Medulloblastoma, cerebellum	+	69,574		C

Primary ID	SAGE Library	Sample	Selection [a]	Tags [b]	Notes [c]	Cluster [d]
138	GSM14735	Pancreas, epithelium, ductal carcinoma cell line	+	31,184		IV
139	GSM14736	Pancreas, epithelium, ductal carcinoma cell line	+	29,495		IV
140	GSM14737	Brain, astrocytoma	+	105,659		C
141	GSM14739	Brain, astrocytoma	+	88,464		C
142	GSM14740	Brain, ependymoma	+	122,655		C
143	GSM14741	Brain, ependymoma	+	120,382		C
144	GSM14742	Brain cortex, oligodendroglioma	+	32,272		C
145	GSM14743	Mammary gland, epithelium, ductal carcinoma	+	72,816		C
146	GSM14744	White blood cells, CD45+CD15+, isolated from a mammary gland ductal carcinoma	+	66,136		D
147	GSM14745	Breast carcinoma myoepithelium	+	81,376		C
148	GSM14746	Breast carcinoma epithelium	+	89,156		C
149	GSM14747	Breast carcinoma myoepithelium	+	37,392		C
150	GSM14748	Vascular endothelium, tumor associated (isolated from mammary gland ductal carcinoma)	+	65,236		D
151	GSM14749	First trimester placenta	+	89,080		N
152	GSM14750	Full-term placenta	+	117,562		N
153	GSM14751	Skin, melanoma	+	26,030		C
154	GSM14752	Prostate epithelium	+	70,944		IV

Primary ID	SAGE Library	Sample	Selection [a]	Tags [b]	Notes [c]	Cluster [d]
155	GSM14753	Breast carcinoma metastasis to lung	+	49,538		C
156	GSM14754	Mammary gland, endothelium	+	33,089		N
157	GSM14755	White blood cells, CD45+CD15+	+	31,970		N
158	GSM14756	Mammary gland	+	58,039		N
159	GSM14757	Breast stroma	+	78,921		N
160	GSM14758	Prostate epithelium, senescent cells	+	70,949		IV
161	GSM14759	Brain, medulloblastoma, cell line	+	66,000		IV
162	GSM14760	Stomach, poorly differentiated carcinoma	+	51,456		C
163	GSM14761	Brain, medulloblastoma	+	84,891		C
164	GSM14762	Brain, ependymoma	+	68,552		C
165	GSM14763	Brain, anaplastic astrocytoma	+	106,942		C
166	GSM14764	Brain, medulloblastoma, cell line	+	66,210		IV
167	GSM14765	Brain, astrocytoma	+	102,362		C
168	GSM14766	Brain, astrocytoma, anaplastic	+	107,172		C
169	GSM14767	Brain, glioblastoma	+	100,496		C
170	GSM14768	Brain, glioblastoma	+	102,261		C
171	GSM14769	Brain, glioblastoma	+	99,061		C
172	GSM14772	Brain, medulloblastoma	+	60,405		C
173	GSM14773	Brain, anaplastic astrocytoma	+	118,642		C

Primary ID	SAGE Library	Sample	Selection [a]	Tags [b]	Notes [c]	Cluster [d]
174	GSM14774	Brain, medulloblastoma with neuroblastic differentiation	+	85,699		C
175	GSM14775	Skin, primary malignant melanoma	+	41,332		C
176	GSM14776	Brain, ependymoma	+	75,215		C
177	GSM14777	Thyroid	+	115,752		N
178	GSM14778	Skin, desmoplastic melanoma metastasis	x		11,392 tags	
179	GSM14779	Brain, medulloblastoma	+	72,077		C
180	GSM14780	Gastric epithelial tissue from the antrum	+	26,619		N
181	GSM14781	Brain desmoplastic medulloblastoma	+	83,506		C
182	GSM14782	Brain desmoplastic medulloblastoma	+	68,158		C
183	GSM14784	Bone marrow	+	36,358		N
184	GSM14785	Lymph node	+	99,084		N
185	GSM14786	Brain, ependymoma	+	83,857		C
186	GSM14787	Brain, anaplastic medulloblastoma	+	57,376		C
187	GSM14788	Brain, medulloblastoma	+	74,158		C
188	GSM14789	Bone marrow, CD34+/CD38-/lin- cells	+	86,377		N
189	GSM14790	Brain, medulloblastoma	+	45,296		C
190	GSM14791	Brain, medulloblastoma	+	32,406		C
191	GSM14792	Brain, large cell medulloblastoma, metastasis	+	59,458		C
192	GSM14793	Brain, medulloblastoma, metastasis	+	88,976		C

Primary ID	SAGE Library	Sample	Selection [a]	Tags [b]	Notes [c]	Cluster [d]
193	GSM14794	Brain, medulloblastoma	+	74,167		C
194	GSM14795	Brain, large cell medulloblastoma	+	67,134		C
195	GSM14796	Brain, substantia nigra	+	42,326		N
196	GSM14797	Breast, invasive ductal carcinoma	+	21,909		C
197	GSM14798	Liver	+	78,257		N
198	GSM14799	Fetal brain	+	308,295		N
199	GSM14801	Breast	+	59,185		N
200	GSM14802	Bone marrow, CD34+/CD38+/lin+ cells	+	81,576		N
201	GSM14804	Lung cell line	+	28,928		IV
202	GSM14805	Lung cell line	+	42,168		IV
203	GSM14806	Lung, well differentiated adenocarcinoma with lymphoplasmatic infiltration	+	35,889		C
204	GSM14807	Lung, poorly differentiated adenocarcinoma with lymphoplasmatic infiltration	+	86,827		C
205	GSM14916	Neonatal BJ foreskin primary fibroblasts	+	57,431		IV
206	GSM14917	Neonatal BJ foreskin primary fibroblasts immortalized by telomerase overexpression	-			
207	GSM14918	AT dermal fibroblasts	+	64,180		IV

Primary ID	SAGE Library	Sample	Selection [a]	Tags [b]	Notes [c]	Cluster [d]
208	GSM14919	AT dermal fibroblasts immortalized by telomerase overexpression	-			
209	GSM16892	Glomerular endothelial cells	+	68,978		N
210	GSM23393	Cultured THP-1 cells, stimulated with PMA	-			
211	GSM23394	Cultured THP-1 cells	x		3,519 tags	
212	GSM26246	Primary umbilical arterial endothelial cell (HUAEC)	+	26,127		IV
213	GSM26247	Primary umbilical vein endothelial cell (HUVEC)	x		13,675 tags	
214	GSM32266	Pulmonary microvascular endothelial cells in culture	+	30,615		IV
215	GSM37211	Adrenal cortex	+	20,859		N
216	GSM37212	Adrenal cortex affected by primary pigmented nodular adrenocortical disease	+	21,638		D
217	GSM37337	Primary bronchial epithelial cells	+	28,577		IV
218	GSM37339	Primary bronchial epithelial cells, stimulated with IL1β and TNFα	-			
219	GSM37341	Primary bronchial epithelial cells, stimulated with heat-inactivated Pseudomonas aeruginosa (strain PAO1)	-			
220	GSM41248	Pulmonary microvascular endothelial cells in culture, exposed to sustained low shear stress	+	31,130		IV
221	GSM43175	Bone marrow CD34+ cells	+	99,488		N

Primary ID	SAGE Library	Sample	Selection [a]	Tags [b]	Notes [c]	Cluster [d]
222	GSM45608	Pulmonary microvascular endothelial cells in culture, exposed to sustained high shear stress	+	24,773		IV
223	GSM48250	Sciatic nerve	x		19,751 tags	
224	GSM48251	Cultured Schwann cells	+	42,280		IV
225	GSM52500	Gastric cardia biopt from a metaplastic Barrett's esophagus patient	+	25,749		D
226	GSM52501	Squamous esophagus biopt from a metaplastic Barrett's esophagus patient	+	50,409		D
227	GSM52502	Metaplastic Barrett's Esophagus biopt	+	46,171		D
228	GSM62240	Primary aortic endothelial cells	+	38,364		IV
229	GSM62241	Primary aortic endothelial cells, exposure to 8h hypoxia	+	40,540		IV
230	GSM62242	Primary aortic endothelial cells, exposure to 24h hypoxia	+	42,305		IV
231	GSM62243	Primary pulmonary artery endothelial cells	+	25,628		IV
232	GSM62244	Primary pulmonary artery endothelial cells, exposure to 8h hypoxia	+	27,581		IV
233	GSM62245	Primary pulmonary artery endothelial cells, exposure to 24h hypoxia	+	42,132		IV
234	GSM66698	HL-60 cells	+	52,160		IV
235	GSM66712	HL-60 cells exposed to 2.45 GHz radiofrequency for 2h	+	51,916		IV

Primary ID	SAGE Library	Sample	Selection [a]	Tags [b]	Notes [c]	Cluster [d]
236	GSM66714	HL-60 cells exposed to 2.45 GHz radiofrequency for 6h	+	51,601		IV
237	GSM73364	Bone marrow, CD15+ myeloid progenitor cells, AML with chromosomal inversion INV(16)	-			
238	GSM73365	Bone marrow, CD15+ myeloid progenitor cells, AML with chromosomal inversion INV(16)	-			
239	GSM73366	Bone marrow, CD15+ myeloid progenitor cells, AML with chromosomal inversion INV(16)	-			
240	GSM73367	Bone marrow, CD15+ myeloid progenitor cells, AML with chromosomal inversion INV(16)	-			
241	GSM73368	Bone marrow, CD15+ myeloid progenitor cells, AML with chromosomal inversion INV(16)	-			
242	GSM73369	Bone marrow, CD15+ myeloid progenitor cells, AML with chromosomal translocation t(8;21)	-			
243	GSM73370	Bone marrow, CD15+ myeloid progenitor cells, AML with chromosomal translocation t(8;21)	-			
244	GSM73371	Bone marrow, CD15+ myeloid progenitor cells, AML with chromosomal translocation t(8;21)	-			
245	GSM73372	Bone marrow, CD15+ myeloid progenitor cells, AML with chromosomal translocation t(8;21)	-			

Primary ID	SAGE Library	Sample	Selection [a]	Tags [b]	Notes [c]	Cluster [d]
246	GSM73373	Bone marrow, CD15+ myeloid progenitor cells, AML with chromosomal translocation t(8;21)	-			
247	GSM73374	Bone marrow, CD15+ myeloid progenitor cells, AML with chromosomal translocation t(9;11)	-			
248	GSM73375	Bone marrow, CD15+ myeloid progenitor cells, AML with chromosomal translocation t(9;11)	-			
249	GSM73376	Bone marrow, CD15+ myeloid progenitor cells, AML with chromosomal translocation t(9;11)	-			
250	GSM73377	Bone marrow, CD15+ myeloid progenitor cells, AML with chromosomal translocation t(9;11)	-			
251	GSM73378	Bone marrow, CD15+ myeloid progenitor cells, AML with chromosomal translocation t(9;11)	-			
252	GSM73379	Bone marrow, CD15+ myeloid progenitor cells, AML with chromosomal translocation t(9;11)	-			
253	GSM73380	Bone marrow, CD15+ myeloid progenitor cells, AML with chromosomal translocation t(9;11)	-			
254	GSM73381	Bone marrow, CD15+ myeloid progenitor cells, AML with chromosomal translocation t(15;17)	-			

Primary ID	SAGE Library	Sample	Selection [a]	Tags [b]	Notes [c]	Cluster [d]
255	GSM73382	Bone marrow, CD15+ myeloid progenitor cells, AML with chromosomal translocation t(15;17)	-			
256	GSM73383	Bone marrow, CD15+ myeloid progenitor cells, AML with chromosomal translocation t(15;17)	-			
257	GSM73384	Bone marrow, CD15+ myeloid progenitor cells, AML with chromosomal translocation t(15;17)	-			
258	GSM73385	Bone marrow, CD15+ myeloid progenitor cells, AML with chromosomal translocation t(15;17)	-			
259	GSM82243	Spermatozoa, pooled sample	+	20,991		N
260	GSM82458	Hippocampus	+	27,936		N
261	GSM82459	Spermatozoa	+	20,184		N
262	GSM85611	Bronchial epithelium	+	83,291		N
263	GSM85616	Bronchial epithelium	+	92,344		N
264	GSM85622	Lung parenchyma, pooled sample	+	65,982		N
265	GSM85623	Lung parenchyma, pooled sample	+	64,300		N
266	GSM110378	Barrett long-term primary cell culture	+	44,885		IV
267	GSM110379	Esophageal adenocarcinoma	+	33,578		C
268	GSM110380	Squamous esophagus long-term primary cell culture	+	43,284		IV
269	GSM110381	Esophageal squamous cell carcinoma	+	24,869		C

Primary ID	SAGE Library	Sample	Selection [a]	Tags [b]	Notes [c]	Cluster [d]
270	GSM112795	Neuroblastoma, primary tumor, stage 4S	+	27,108		C
271	GSM112797	Neuroblastoma, primary tumor, stage 4S	+	28,062		C
272	GSM112807	Neuroblastoma, primary tumor, stage 4S	+	26,420		C
273	GSM112808	Neuroblastoma, primary tumor, stage 4S	+	28,677		C
274	GSM112809	Neuroblastoma, primary tumor, stage 4S	+	25,095		C
275	GSM112810	Neuroblastoma, primary tumor, stage 4	x		19,599 tags	
276	GSM112812	Neuroblastoma, primary tumor, stage 4	+	29,395		C
277	GSM112813	Neuroblastoma, primary tumor, stage 4	+	27,197		C
278	GSM112814	Neuroblastoma, cell line, IMR-5	x		8,568 tags	
279	GSM113361	Liver myofibroblast	x		13,009 tags	
280	GSM113362	Liver stellate cells 0 days	x		15,328 tags	
281	GSM113363	Liver stellate cells 15 days	+	27,550		IV
282	GSM118371	CD14+ monocytes isolated from peripheral blood	+	57,666		N
283	GSM125335	Bronchial brushings, current smoker	+	92,992		N
284	GSM125336	Bronchial brushings, current smoker	+	80,856		N
285	GSM125337	Bronchial brushings, current smoker	+	87,564		N
286	GSM125338	Bronchial brushings, current smoker	+	81,495		N
287	GSM125339	Bronchial brushings, current smoker	+	99,934		N
288	GSM125340	Bronchial brushings, current smoker	+	171,729		N

Primary ID	SAGE Library	Sample	Selection [a]	Tags [b]	Notes [c]	Cluster [d]
289	GSM125341	Bronchial brushings, current smoker	+	151,918		N
290	GSM125342	Bronchial brushings, current smoker	+	173,260		N
291	GSM125343	Bronchial brushings, former smoker	+	151,454		N
292	GSM125344	Bronchial brushings, former smoker	+	221,528		N
293	GSM125345	Bronchial brushings, former smoker	+	162,278		N
294	GSM125346	Bronchial brushings, former smoker	+	157,360		N
295	GSM125347	Bronchial brushings, former smoker	+	91,250		N
296	GSM125348	Bronchial brushings, former smoker	+	86,436		N
297	GSM125349	Bronchial brushings, former smoker	+	89,288		N
298	GSM125350	Bronchial brushings, former smoker	+	154,657		N
299	GSM125351	Bronchial brushings, former smoker	+	142,314		N
300	GSM125352	Bronchial brushings, former smoker	+	130,274		N
301	GSM125353	Bronchial brushings, former smoker	+	81,214		N
302	GSM125354	Bronchial brushings, former smoker	+	154,446		N
303	GSM125355	Bronchial brushings, never smoker	+	137,755		N
304	GSM125356	Bronchial brushings, never smoker	+	127,203		N
305	GSM125357	Bronchial brushings, never smoker	+	131,731		N
306	GSM125358	Bronchial brushings, never smoker	+	138,933		N
307	GSM135388	Skeletal muscle, pretraining young men	+	54,959		N

Primary ID	SAGE Library	Sample	Selection [a]	Tags [b]	Notes [c]	Cluster [d]
308	GSM135389	Skeletal muscle, 5 days training young men	+	59,751		N
309	GSM135390	Skeletal muscle, 6 wk training young men	+	56,035		N
310	GSM135391	Skeletal muscle, 12 wk training young men	+	57,183		N
311	GSM135392	Skeletal muscle, detraining young men	+	64,654		N
312	GSM136119	LPS-stimulated CD14+ monocytes	+	35,955		D
313	GSM136120	M-CSF-induced macrophages	+	53,983		D
314	GSM136121	GM-CSF-induced macrophages	+	57,482		D
315	GSM136123	Monocyte-derived immature dendritic cells	+	58,651		IV
316	GSM136124	Monocyte-derived mature dendritic cells	+	31,810		IV
317	GSM136194	Naive CD4 T Cells (CD45RA+)	+	50,408		N
318	GSM136195	Cord blood-derived activated Th1 cells	+	32,163		N
319	GSM136196	Cord blood-derived activated Th2 cells	+	32,224		N
320	GSM136197	CD56+ NK cells	+	34,809		N
321	GSM136198	CD8+ T cells	+	50,993		N
322	GSM136199	CD19+ B cells	+	53,195		N
323	GSM154136	Hippocampus; Alzheimer-affected, APOE3/3	+	78,088		D
324	GSM154137	Hippocampus; control, APOE3/3	+	70,435		N
325	GSM154138	Hippocampus; Alzheimer-affected, APOE3/4	+	72,055		D
326	GSM154139	Hippocampus; Alzheimer-affected, APOE4/4	+	67,944		D

Primary ID	SAGE Library	Sample	Selection [a]	Tags [b]	Notes [c]	Cluster [d]
327	GSM180669	Lymphocytes from Down syndrome children (pooled samples)	-			
328	GSM180670	Lymphocytes from children 1-4 years old (pooled samples)	+	40,324		N
329	GSM194375	Non-small cell lung cancer: squamous cell carcinoma-in-situ	+	161,565		C
330	GSM194376	Non-small cell lung cancer: squamous cell carcinoma-in-situ	+	159,507		C
331	GSM194377	Non-small cell lung cancer: squamous cell carcinoma-in-situ	+	199,977		C
332	GSM194378	Non-small cell lung cancer: squamous cell carcinoma-in-situ	+	172,948		C
333	GSM194379	Non-small cell lung cancer: squamous cell carcinoma-in-situ	+	208,836		C
334	GSM194380	Non-small cell lung cancer: invasive squamous cell carcinoma	+	149,646		C
335	GSM194381	Non-small cell lung cancer: invasive squamous cell carcinoma	+	151,041		C
336	GSM194382	Non-small cell lung cancer: invasive squamous cell carcinoma	+	207,897		C
337	GSM194383	Non-small cell lung cancer: invasive squamous cell carcinoma	+	176,033		C
338	GSM194384	Non-small cell lung cancer: invasive squamous cell carcinoma	+	152,390		C
339	GSM194385	Non-small cell lung cancer: invasive squamous cell carcinoma	+	148,851		C
340	GSM194386	Metaplastic bronchial epithelium	+	200,451		D
341	GSM194387	Dysplastic bronchial epithelium	+	155,046		D
342	GSM194647	Oral brushing	+	168,569		N
343	GSM194648	Oral brushing	+	126,241		N
344	GSM194649	Oral brushing	+	176,557		N
345	GSM194650	Oral biopsy	+	177,035		N

Primary ID	SAGE Library	Sample	Selection [a]	Tags [b]	Notes [c]	Cluster [d]
346	GSM194651	Oral biopsy	+	156,764		N
347	GSM194652	Oral biopsy	+	167,109		N
348	GSM209897	In vitro cultured Schwann cells from patient with duplication of PMP22	-			
349	GSM209898	In vitro cultured Schwann cells from patient with missense mutation of NDRG1 (R148X)	-			
350	GSM237078	Endemic Kaposi's sarcoma tissue	+	48,390		D
351	GSM264073	Resting derived macrophages	+	28,150		IV
352	GSM264106	Leishmania major infected derived macrophages	-			
353	GSM296374	Lung biopsy	+	69,896		N
354	GSM296376	Lung biopsy	+	112,860		N
355	GSM296377	Lung biopsy	+	107,410		N
356	GSM296379	Lung biopsy	+	90,530		N
357	GSM296381	Lung biopsy	+	57,426		N
358	GSM296382	Lung biopsy	+	96,827		N
359	GSM296385	Lung biopsy	+	86,381		N
360	GSM296387	Lung biopsy	+	97,711		N
361	GSM296391	Lung biopsy	+	59,395		N
362	GSM296392	Lung biopsy	+	43,222		N

Primary ID	SAGE Library	Sample	Selection [a]	Tags [b]	Notes [c]	Cluster [d]
363	GSM296393	Lung biopsy	+	31,772		N
364	GSM296644	Lung biopsy	+	99,887		N
365	GSM301092	Lung parenchyma	+	55,653		N
366	GSM301093	Lung parenchyma	+	78,783		N
367	GSM301094	Huh7 stably expressing S33Y β-catenin	-			
368	GSM301095	Huh7 Cells with PCI-Neo stable integration into genome	-			
369	GSM303325	Margins of laryngeal squamous cell carcinoma (LSCC)	+	102,202		C
370	GSM303340	Laryngeal Squamous Cell Carcinoma (LSCC)	+	99,578		C
371	GSM303349	Laryngeal Squamous Cell Carcinoma (LSCC)	+	46,928		C
372	GSM311354	CD15+ myeloid progenitor cells	+	104,683		N
373	GSM383686	Fetal cortex astrocytes	+	51,361		N
374	GSM383688	Astrocytoma grade II	+	114,111		C
375	GSM383690	Astrocytoma grade II	+	106,201		C
376	GSM383691	Astrocytoma grade II	+	128,130		C
377	GSM383692	Astrocytoma grade II	+	108,036		C
378	GSM383693	Astrocytoma grade II	+	115,879		C
379	GSM383696	Astrocytoma grade III	+	100,026		C
380	GSM383697	Astrocytoma grade III	+	108,263		C
381	GSM383698	Astrocytoma grade III	+	107,758		C

Primary ID	SAGE Library	Sample	Selection [a]	Tags [b]	Notes [c]	Cluster [d]
382	GSM383699	Astrocytoma grade III	+	102,932		C
383	GSM383700	Astrocytoma grade III	+	99,886		C
384	GSM383702	Astrocytoma grade III	+	109,854		C
385	GSM383703	Astrocytoma grade III	+	95,975		C
386	GSM383709	Ependymoma	+	56,275		C
387	GSM383710	Ependymoma	+	46,564		C
388	GSM383713	Ependymoma	+	73,696		C
389	GSM383717	Ependymoma	+	52,126		C
390	GSM383720	Glioblastoma multiforme	+	59,109		C
391	GSM383722	Glioblastoma multiforme	+	124,316		C
392	GSM383723	Glioblastoma multiforme	+	49,331		C
393	GSM383724	Glioblastoma multiforme	+	88,876		C
394	GSM383726	Glioblastoma multiforme, pooled sample	+	56,347		C
395	GSM383727	Glioblastoma multiforme	+	101,004		C
396	GSM383730	Glioblastoma multiforme, cell line	+	110,579		IV
397	GSM383731	Glioblastoma multiforme, cell line	+	115,115		IV
398	GSM383733	Glioblastoma multiforme, cell line	+	56,807		IV
399	GSM383734	Glioblastoma multiforme, cell line	+	66,726		IV
400	GSM383735	Glioblastoma multiforme, cell line	+	108,340		IV

Primary ID	SAGE Library	Sample	Selection [a]	Tags [b]	Notes [c]	Cluster [d]
401	GSM383736	Glioblastoma multiforme, cell line	+	60,242		IV
402	GSM383737	Glioblastoma multiforme, short term culture	+	46,615		IV
403	GSM383738	Glioblastoma multiforme, cell line	+	71,574		IV
404	GSM383753	Medulloblastoma	+	61,698		C
405	GSM383759	Medulloblastoma cell line	+	62,435		IV
406	GSM383760	Medulloblastoma cell line	+	97,271		IV
407	GSM383763	Medulloblastoma cell line	+	43,836		IV
408	GSM383765	Medulloblastoma cell line	+	94,870		IV
409	GSM383766	Medulloblastoma cell line	+	80,786		IV
410	GSM383767	Benign meningioma	+	69,251		C
411	GSM383768	Benign meningioma	+	54,647		C
412	GSM383769	Malignant meningioma	+	48,706		C
413	GSM383770	Malignant meningioma	+	44,852		C
414	GSM383771	Malignant meningioma	+	59,784		C
415	GSM383772	Cerebellum	+	50,244		N
416	GSM383775	Cortex, pooled sample	+	62,329		N
417	GSM383776	Brain, leptomeninges	+	72,469		N
418	GSM383781	Oligodendroglioma	+	27,844		C
419	GSM383783	Breast carcinoma myoepithelium	+	51,941		C

Primary ID	SAGE Library	Sample	Selection [a]	Tags [b]	Notes [c]	Cluster [d]
420	GSM383784	Mammary gland myofibroblasts	+	65,061		N
421	GSM383785	Invasive breast cancer myofibroblasts	+	73,786		C
422	GSM383786	Invasive breast cancer myofibroblasts	+	63,383		C
423	GSM383787	Breast stroma, ductal carcinoma in situ associated	+	56,772		D
424	GSM383788	Breast stroma, tumor associated	+	67,786		D
425	GSM383790	Mammary gland, ductal carcinoma	+	67,003		C
426	GSM383791	Mammary gland, ductal carcinoma in situ	+	50,692		C
427	GSM383793	Mammary gland, ductal carcinoma in situ	+	60,138		C
428	GSM383794	Mammary gland, ductal carcinoma in situ	+	42,892		C
429	GSM383796	Mammary gland, ductal carcinoma	+	63,960		C
430	GSM383797	Mammary gland, ductal carcinoma	+	60,208		C
431	GSM383798	Breast carcinoma MCF7 cell line	+	59,639		IV
432	GSM383799	Breast carcinoma cell line	+	60,623		IV
433	GSM383800	Breast carcinoma cell line	+	59,949		IV
434	GSM383801	Breast carcinoma cell line	+	47,016		IV
435	GSM383802	Breast carcinoma cell line	+	46,438		IV
436	GSM383803	Breast carcinoma cell line	+	40,159		IV
437	GSM383804	Breast carcinoma cell line	+	38,447		IV
438	GSM383805	Breast carcinoma cell line	+	39,868		IV

Primary ID	SAGE Library	Sample	Selection [a]	Tags [b]	Notes [c]	Cluster [d]
439	GSM383806	Breast carcinoma cell line	+	32,070		IV
440	GSM383807	Mammary gland, ductal carcinoma in situ	+	66,018		C
441	GSM383808	Mammary gland, ductal carcinoma in situ	+	57,380		C
442	GSM383811	Breast carcinoma	+	66,475		C
443	GSM383812	Mammary gland, ductal carcinoma	+	65,780		C
444	GSM383813	Breast carcinoma	+	73,293		C
445	GSM383814	Mammary gland, ductal carcinoma	+	39,349		C
446	GSM383815	Mammary gland, ductal carcinoma	+	57,895		C
447	GSM383816	Breast cancer	+	50,127		C
448	GSM383817	Mammary gland, lobular/ductal carcinoma	+	52,477		C
449	GSM383818	Breast cancer	+	52,319		C
450	GSM383819	Mammary gland, ductal carcinoma	+	52,773		C
451	GSM383820	Mammary gland, ductal carcinoma	+	54,055		C
452	GSM383821	Breast cancer	+	75,125		C
453	GSM383823	Breast carcinoma	+	67,742		C
454	GSM383825	Breast cancer	+	78,771		C
455	GSM383826	Breast juvenile fibroadenoma	+	55,429		D
456	GSM383828	Breast carcinoma, metastasis	+	60,214		C
457	GSM383831	Mammary epithelium	+	50,393		N

Primary ID	SAGE Library	Sample	Selection [a]	Tags [b]	Notes [c]	Cluster [d]
458	GSM383832	Mammary epithelium, CD24+ cells	+	41,420		N
459	GSM383833	Mammary epithelium, CD24+ cells	+	41,457		N
460	GSM383834	Mammary epithelium, CD24+ cells	+	76,463		N
461	GSM383835	Mammary epithelial stem cells, CD44+	+	56,003		N
462	GSM383836	Mammary epithelial stem cells, CD44+	+	80,453		N
463	GSM383837	Mammary epithelium, Muc1+ cells	+	71,700		N
464	GSM383838	Mammary epithelium, Muc1+ cells	+	76,216		N
465	GSM383839	Mammary epithelium	+	34,544		N
466	GSM383840	Mammary myoepithelium, CD10+ cells	+	68,651		N
467	GSM383842	Mammary myoepithelium, CD10+ cells	+	63,975		N
468	GSM383843	Mammary myoepithelium, CD10+ cells	+	76,161		N
469	GSM383844	Spontaneously immortalized mammary myoepithelial cell line	-			
470	GSM383847	Breast stroma	+	50,435		N
471	GSM383848	Breast cancer	+	59,449		C
472	GSM383849	Cartilage chondrosarcoma cell line	+	33,071		IV
473	GSM383850	Cartilage chondrosarcoma	+	93,558		C
474	GSM383851	Cartilage chondrosarcoma cell line	+	74,878		IV
475	GSM383852	Cartilage chondrosarcoma cell line	+	77,649		IV
476	GSM383853	Cartilage chondrosarcoma cell line	+	68,010		IV

Primary ID	SAGE Library	Sample	Selection [a]	Tags [b]	Notes [c]	Cluster [d]
477	GSM383854	Cartilage chondrosarcoma metastasis cell line	+	37,739		IV
478	GSM383855	Cartilage chondrosarcoma metastasis cell line	+	85,876		IV
479	GSM383856	Cartilage chondrosarcoma metastasis cell line	+	109,685		IV
480	GSM383857	Cartilage chondrosarcoma metastasis cell line	+	108,238		IV
481	GSM383858	Cartilage chondrosarcoma metastasis cell line	+	88,431		IV
482	GSM383859	Colon adenocarcinoma	+	55,666		C
483	GSM383861	Colon adenocarcinoma, cell line	+	60,509		IV
484	GSM383864	Colon adenocarcinoma, cell line	+	60,932		IV
485	GSM383865	Colon carcinoma, cell line	+	79,051		IV
486	GSM383866	Colon carcinoma, cell line	+	79,282		IV
487	GSM383867	Colon carcinoma, cell line	+	79,014		IV
488	GSM383868	Colon carcinoma, cell line	+	80,396		IV
489	GSM383869	Colonic epithelium	+	49,593		N
490	GSM383872	Embryonic stem cell line BG01	+	201,197		IV
491	GSM383873	Embryonic stem cell line H1	+	275,844		IV
492	GSM383874	Embryonic stem cell line H1	+	218,182		IV
493	GSM383875	Embryonic stem cell line H13	+	220,990		IV
494	GSM383876	Embryonic stem cell line H14	+	211,993		IV
495	GSM383877	Embryonic stem cell line H7	+	272,361		IV

Primary ID	SAGE Library	Sample	Selection [a]	Tags [b]	Notes [c]	Cluster [d]
496	GSM383878	Embryonic stem cell line H9	+	151,189		IV
497	GSM383879	Embryonic stem cell line H9	+	400,906		IV
498	GSM383880	Embryonic stem cell line HES3	+	204,974		IV
499	GSM383881	Embryonic stem cell line HES4	+	209,114		IV
500	GSM383882	Embryonic stem cell line HSF6	+	224,051		IV
501	GSM383884	Esophagus adenocarcinoma	+	73,771		C
502	GSM383885	Esophagus pre-cancer (high grade dysplasia)	+	78,591		D
503	GSM383886	Esophagus pre-cancer (low grade dysplasia)	+	74,892		D
504	GSM383887	Esophagus	+	75,629		N
505	GSM383888	Eye lens	+	85,834		N
506	GSM383892	Gallbladder adenocarcinoma	+	81,331		C
507	GSM383893	Gallbladder tubular adenocarcinoma	+	66,933		C
508	GSM383894	Gallbladder tubular adenocarcinoma	+	78,498		C
509	GSM383895	Gallbladder	+	85,559		N
510	GSM383897	Kidney, transitional cell carcinoma	+	100,209		C
511	GSM383898	Kidney, embryonic cell line 293, induced cells	+	23,445		IV
512	GSM383900	Kidney glomeruli	+	48,904		N
513	GSM383901	Kidney	+	40,790		N
514	GSM383902	Leukocytes	+	47,799		N

Primary ID	SAGE Library	Sample	Selection [a]	Tags [b]	Notes [c]	Cluster [d]
515	GSM383903	Liver cholangiocarcinoma metastasis	+	60,300		C
516	GSM383904	Liver adenocarcinoma	+	46,720		C
517	GSM383905	Liver carcinoma cell line	+	54,695		IV
518	GSM383906	Liver cholangiocarcinoma cell line	+	52,859		IV
519	GSM383907	Liver	+	66,056		N
520	GSM383908	Lung adenocarcinoma	+	36,132		C
521	GSM383911	Lung	+	88,468		N
522	GSM383914	Lung, tumor associated (focal fibrosis and chronic inflammation)	+	98,937		D
523	GSM383915	Lymph node, B cell lymphoma	+	71,993		C
524	GSM383921	Muscle, rhabdomyosarcoma	+	61,235		C
525	GSM383925	Ovary, carcinoma, cell line	+	21,327		IV
526	GSM383926	Ovary, clear cell carcinoma	+	31,070		C
527	GSM383927	Ovary, cystadenoma cell line immortalized with SV40	-			
528	GSM383928	Ovary preneoplasia cell line	+	76,019		IV
529	GSM383929	Ovary, surface epithelium immortalized with SV40	-			
530	GSM383930	Ovary epithelium, short term culture	+	47,597		IV
531	GSM383933	Pancreatic adenocarcinoma, cell line	+	37,606		IV
532	GSM383934	Pancreatic adenocarcinoma, cell line	+	24,716		IV

Primary ID	SAGE Library	Sample	Selection [a]	Tags [b]	Notes [c]	Cluster [d]
533	GSM383937	Pancreas	+	21,510		N
534	GSM383938	Pancreatic ductal epithelium, short term culture	+	32,222		IV
535	GSM383939	Pancreatic ductal epithelium, short term culture	+	31,983		IV
536	GSM383940	Bone, chondrosarcoma	+	83,566		C
537	GSM383946	Whole body, fetal	+	101,557		N
538	GSM383948	Prostatic adenocarcinoma, cell line	+	22,224		IV
539	GSM383949	Prostatic adenocarcinoma	+	64,854		IV
540	GSM383952	Prostatic carcinoma cell line	+	30,230		IV
541	GSM383956	Prostatic carcinoma cell line	+	43,317		IV
542	GSM383962	Prostate	+	59,166		N
543	GSM383963	Retina, macula	+	101,155		N
544	GSM383965	Central retina	+	103,499		N
545	GSM383968	Peripheral retina	+	126,405		N
546	GSM383970	Retinoblastoma	+	88,438		C
547	GSM383971	Macular retinal pigment epithelium	+	77,071		N
548	GSM383972	Choroid retinal pigment epithelium	+	89,667		N
549	GSM383973	Synovial fibroblasts from rheumatoid arthritis	+	42,382		D
550	GSM383994	Stomach adenocarcinoma	+	46,727		C
551	GSM383995	Stomach/lower esophagus adenocarcinoma	+	93,546		C

Primary ID	SAGE Library	Sample	Selection [a]	Tags [b]	Notes [c]	Cluster [d]
552	GSM383996	Gastroesophageal junction adenocarcinoma	+	58,705		C
553	GSM383998	Gastroesophageal junction adenocarcinoma	+	69,547		C
554	GSM384002	Stomach	+	45,845		N
555	GSM384003	Stomach	+	73,288		N
556	GSM384006	Testis, embyronal carcinoma	+	66,349		C
557	GSM384007	Thyroid, follicular adenoma	+	101,549		C
558	GSM384008	Thyroid, follicular carcinoma	+	124,310		C
559	GSM384010	Mixture of cancer cell lines	+	51,467		IV
560	GSM384012	Endometrium	+	21,828		N
561	GSM384016	Vascular endothelium, hemangioma, benign hyperplasia	+	75,590		D
562	GSM384017	Vascular endothelium	+	56,984		N
563	GSM384020	Vascular endothelium, short term culture	+	57,245		IV
564	GSM384024	White blood cells, CD45+, isolated from a mammary gland carcinoma	x		18,741 tags	
565	GSM384025	White blood cells, CD45+, isolated from a mammary gland carcinoma	+	34,365		C
566	GSM384026	White blood cell, lung macrophage	+	51,867		N
567	GSM384027	White blood cell, monocyte-depleted mononuclear cells	+	53,781		N
568	GSM384028	White blood cell, monocyte-depleted mononuclear cells	+	51,615		N

Primary ID	SAGE Library	Sample	Selection [a]	Tags [b]	Notes [c]	Cluster [d]
569	GSM384029	White blood cell, monocyte	+	99,910		N
570	GSM384030	White blood cell, monocyte	+	101,142		N
571	GSM384031	White blood cell, monocyte	+	98,778		N
572	GSM384032	White blood cell, monocyte	+	101,648		N
573	GSM384033	White blood cell, monocyte	+	54,415		N
574	GSM384035	White blood cell, plaque macrophage	+	102,847		N
575	GSM384036	White blood cell, plaque macrophage	+	50,294		N
576	GSM389906	Total blood, pooled sample	+	29,779		N
577	GSM389907	Total blood during EPO treatment, pooled sample	+	30,798		D
578	GSM389908	Total blood after EPO treatment, pooled sample	+	29,424		D
579	GSM549178	Bone marrow CD34+ cells	+	61,289		N
580	GSM549179	Bone marrow CD34+ cells cultured for 12 hours under myeloid differentiation stimulus	+	62,057		IV
581	GSM549180	Bone marrow CD34+ cells cultured for 40 hours under myeloid differentiation stimulus	+	63,022		IV

Indexes (GSM#) represent GEO database accession numbers for SAGE libraries (one accession number selected for redundant entries). Entries are sorted according to these indexes. When exclusion duplicate GEO database entries, one with higher (later generated) index was generally excluded.

[a] (+), selected for analysis; (**-**), genetically modified or overwise altered species or cell cultures; (**x**), SAGE libraries with a total number of reliable tags ≤ 20,000 or with complete dataset unavailable.

[b] Number of tags subjected for analysis (with $(A)_{10}$ tags excluded).

[c] Number of tags for SAGE libraries with a total number of reliable tags ≤ 20,000 is shown; (1 tag (-)), tags detected once in a library are not available via GEO database; Asterisks (*) indicate SAGE libraries constructed by author *et al*. and subjected to 'clean-up' procedure [10].

[d] Clusters: C, cancer tissue; N, normal tissue and cells; IV, cells cultured in vitro; D, non-tumorous disease tissue and cells.

Supplemental Table 2. Key properties of established and candidate imprinted gene subset within the SAGE libraries.

ID [a]	Primary ID [b]	SAGE Library	Sample	Sum [c]	Average [d]	Max [e]
			Cancer			
1	9	GSM670	Breast tumor	6,274.28	36.27	2,440.00
2	10	GSM671	Metastasis from breast tumor	5,196.11	30.04	1,359.32
3	19	GSM686	Prostate tumor	8,593.52	49.67	1,327.69
4	20	GSM687	Breast, ductal carcinoma in situ	6,032.85	34.87	920.68
5	21	GSM688	Breast, ductal carcinoma in situ	19,968.16	115.42	15,538.48
6	22	GSM689	Primary tumor, well-differentiated oligodendroglioma	8,572.85	49.55	2,916.90
7	23	GSM690	Brain, medulloblastoma grade IV	8,887.52	51.37	1,926.49
8	29	GSM696	Brain, Duke glioblastoma multiforme primary tumor	11,851.70	68.51	6,764.21
9	30	GSM697	Brain, anaplastic astrocytoma, primary tumor	4,424.78	25.58	915.47
10	31	GSM698	Pilocytic astrocytoma, primary tumor	7,340.33	42.43	1,818.84
11	32	GSM699	Brain, astrocytoma	5,150.98	29.77	1,207.82
12	33	GSM700	Brain, glioblastoma multiforme	10,579.06	61.15	3,396.44
13	34	GSM701	Brain, glioblastoma multiforme	14,617.13	84.49	5,555.83
14	52	GSM723	Prostate, advanced tumor	5,258.81	30.40	1,694.99
15	54	GSM726	Ovary cystadenoma	9,464.44	54.71	5,285.34
16	55	GSM727	Abdominal peritoneum, primary mesothelioma (pure epithelial)	7,155.89	41.36	3,250.09
17	62	GSM735	Primary ovarian tumor, serous adenocarcinoma	13,381.88	77.35	8,960.66
18	63	GSM736	Primary ovarian tumor, serous adenocarcinoma	10,139.44	58.61	2,694.14

ID [a]	Primary ID [b]	SAGE Library	Sample	Sum [c]	Average [d]	Max [e]
19	64	GSM737	Primary ovarian tumor, serous adenocarcinoma	10,849.16	62.71	3,040.15
20	66	GSM740	Prostate, adenocarcinoma	6,268.01	36.23	1,379.27
21	68	GSM743	Pancreas tumor	7,516.80	43.45	2,476.12
22	69	GSM744	Pancreas tumor	7,055.66	40.78	1,875.91
23	75	GSM756	Colon adenocarcinoma	12,954.69	74.88	5,814.31
24	76	GSM757	Stomach, primary gastric cancer, gastroesophageal junction adenocarcinoma	5,026.19	29.05	1,336.27
25	81	GSM765	Brain, glioblastoma multiforme primary tumor	7,573.43	43.78	2,362.65
26	86	GSM792	Brain, ependymoblastoma, primary tumor	14,817.61	85.65	5,828.45
27	97	GSM1498	Brain, glioblastoma multiforme (glioma)	9,149.12	52.89	2,842.13
28	101	GSM1516	Hemangioma tumor	22,622.41	130.77	12,500.82
29	104	GSM1730	Breast, ductal carcinoma in situ	29,575.98	170.96	25,125.63
30	105	GSM1731	Breast, ductal carcinoma in situ	23,346.84	134.95	18,746.14
31	106	GSM1733	Mammary gland, ductal invasive in situ carcinoma	19,126.03	110.56	14,594.68
32	108	GSM2384	Brain, ependymoma	13,069.55	75.55	6,846.85
33	109	GSM2385	Primary gastric cancer	4,586.58	26.51	907.92
34	111	GSM2408	Brain, ependymoma	10,688.68	61.78	2,358.36
35	112	GSM2443	Brain, astrocytoma	6,935.70	40.09	1,072.79
36	113	GSM2451	Brain, astrocytoma	13,936.74	80.56	8,833.51
37	115	GSM2578	Brain, astrocytoma	6,004.58	34.71	1,411.15

ID [a]	Primary ID [b]	SAGE Library	Sample	Sum [c]	Average [d]	Max [e]
38	127	GSM7800	Primary gastric cancer, poorly differentiated (scirrhous type)	3,413.27	19.73	636.37
39	128	GSM8505	Primary gastric cancer	5,723.40	33.08	1,501.22
40	129	GSM8867	Primary gastric cancer	4,858.71	28.09	1,329.27
41	134	GSM14731	Medulloblastoma, cerebellum	10,363.98	59.91	3,160.92
42	135	GSM14732	Medulloblastoma, cerebellum	6,834.14	39.50	1,594.63
43	136	GSM14733	Medulloblastoma, cerebellum	5,897.02	34.09	1,887.98
44	137	GSM14734	Medulloblastoma, cerebellum	4,081.98	23.60	804.90
45	140	GSM14737	Brain, astrocytoma	5,867.93	33.92	1,523.77
46	141	GSM14739	Brain, astrocytoma	6,262.43	36.20	1,051.28
47	142	GSM14740	Brain, ependymoma	7,533.33	43.55	1,010.97
48	143	GSM14741	Brain, ependymoma	5,391.17	31.16	614.71
49	144	GSM14742	Brain cortex, oligodendroglioma	6,414.23	37.08	1,146.50
50	145	GSM14743	Mammary gland, epithelium, ductal carcinoma	10,615.80	61.36	5,987.70
51	147	GSM14745	Breast carcinoma myoepithelium	8,909.26	51.50	4,030.67
52	148	GSM14746	Breast carcinoma epithelium	14,659.70	84.74	8,401.00
53	149	GSM14747	Breast carcinoma myoepithelium	8,317.29	48.08	2,192.98
54	153	GSM14751	Skin, melanoma	4,072.22	23.54	2,497.12
55	155	GSM14753	Breast carcinoma metastasis to lung	23,941.22	138.39	20,247.08
56	162	GSM14760	Stomach, poorly differentiated carcinoma	4,081.16	23.59	1,340.95
57	163	GSM14761	Brain, medulloblastoma	6,573.13	37.99	1,260.44

ID [a]	Primary ID [b]	SAGE Library	Sample	Sum [c]	Average [d]	Max [e]
58	164	GSM14762	Brain, ependymoma	6,797.76	39.29	1,064.89
59	165	GSM14763	Brain, anaplastic astrocytoma	8,518.64	49.24	2,234.86
60	167	GSM14765	Brain, astrocytoma	7,336.71	42.41	2,198.08
61	168	GSM14766	Brain, astrocytoma, anaplastic	5,887.73	34.03	1,147.69
62	169	GSM14767	Brain, glioblastoma	10,020.30	57.92	2,617.02
63	170	GSM14768	Brain, glioblastoma	6,590.98	38.10	1,975.34
64	171	GSM14769	Brain, glioblastoma	7,076.45	40.90	1,625.26
65	172	GSM14772	Brain, medulloblastoma	6,290.87	36.36	1,622.38
66	173	GSM14773	Brain, anaplastic astrocytoma	7,198.13	41.61	2,056.61
67	174	GSM14774	Brain, medulloblastoma with neuroblastic differentiation	8,751.56	50.59	2,368.76
68	175	GSM14775	Skin, primary malignant melanoma	2,612.99	15.10	653.25
69	176	GSM14776	Brain, ependymoma	10,197.43	58.94	3,589.71
70	179	GSM14779	Brain, medulloblastoma	6,437.56	37.21	1,637.14
71	181	GSM14781	Brain desmoplastic medulloblastoma	18,106.48	104.66	11,244.70
72	182	GSM14782	Brain desmoplastic medulloblastoma	7,350.57	42.49	2,861.00
73	185	GSM14786	Brain, ependymoma	9,933.58	57.42	2,730.84
74	186	GSM14787	Brain, anaplastic medulloblastoma	4,967.23	28.71	732.01
75	187	GSM14788	Brain, medulloblastoma	7,039.02	40.69	1,254.08
76	189	GSM14790	Brain, medulloblastoma	10,486.58	60.62	3,510.24
77	190	GSM14791	Brain, medulloblastoma	4,999.07	28.90	1,296.06

ID [a]	Primary ID [b]	SAGE Library	Sample	Sum [c]	Average [d]	Max [e]
78	191	GSM14792	Brain, large cell medulloblastoma, metastasis	5,331.49	30.82	1,429.58
79	192	GSM14793	Brain, medulloblastoma, metastasis	5,799.32	33.52	1,876.91
80	193	GSM14794	Brain, medulloblastoma	11,366.24	65.70	4,489.87
81	194	GSM14795	Brain, large cell medulloblastoma	4,736.80	27.38	1,042.69
82	196	GSM14797	Breast, invasive ductal carcinoma	5,751.06	33.24	1,917.02
83	203	GSM14806	Lung, well differentiated adenocarcinoma with lymphoplasmatic infiltration	4,569.65	26.41	445.82
84	204	GSM14807	Lung, poorly differentiated adenocarcinoma with lymphoplasmatic infiltration	4,146.18	23.97	460.69
85	267	GSM110379	Esophageal adenocarcinoma	13,639.88	78.84	9,649.18
86	269	GSM110381	Esophageal squamous cell carcinoma	11,339.42	65.55	4,342.76
87	270	GSM112795	Neuroblastoma, primary tumor, stage 4S	8,189.46	47.34	1,992.03
88	271	GSM112797	Neuroblastoma, primary tumor, stage 4S	9,372.10	54.17	1,603.59
89	272	GSM112807	Neuroblastoma, primary tumor, stage 4S	11,884.94	68.70	3,444.36
90	273	GSM112808	Neuroblastoma, primary tumor, stage 4S	25,944.14	149.97	17,365.83
91	274	GSM112809	Neuroblastoma, primary tumor, stage 4S	22,633.99	130.83	11,595.94
92	276	GSM112812	Neuroblastoma, primary tumor, stage 4	18,914.78	109.33	12,349.04
93	277	GSM112813	Neuroblastoma, primary tumor, stage 4	5,588.85	32.31	992.76
94	329	GSM194375	Non-small cell lung cancer: squamous cell carcinoma-in-situ	15,715.04	90.84	11,679.51
95	330	GSM194376	Non-small cell lung cancer: squamous cell carcinoma-in-situ	13,629.50	78.78	10,369.45

ID [a]	Primary ID [b]	SAGE Library	Sample	Sum [c]	Average [d]	Max [e]
96	331	GSM194377	Non-small cell lung cancer: squamous cell carcinoma-in-situ	18,122.08	104.75	14,601.68
97	332	GSM194378	Non-small cell lung cancer: squamous cell carcinoma-in-situ	7,013.67	40.54	3,446.12
98	333	GSM194379	Non-small cell lung cancer: squamous cell carcinoma-in-situ	16,572.81	95.80	12,713.33
99	334	GSM194380	Non-small cell lung cancer: invasive squamous cell carcinoma	14,895.15	86.10	9,843.23
100	335	GSM194381	Non-small cell lung cancer: invasive squamous cell carcinoma	14,817.17	85.65	10,374.67
101	336	GSM194382	Non-small cell lung cancer: invasive squamous cell carcinoma	13,929.97	80.52	9,201.67
102	337	GSM194383	Non-small cell lung cancer: invasive squamous cell carcinoma	13,310.00	76.94	8,981.27
103	338	GSM194384	Non-small cell lung cancer: invasive squamous cell carcinoma	6,877.09	39.75	2,093.31
104	339	GSM194385	Non-small cell lung cancer: invasive squamous cell carcinoma	9,553.18	55.22	5,448.40
105	369	GSM303325	Margins of laryngeal squamous cell carcinoma (LSCC)	9,579.07	55.37	2,201.52
106	370	GSM303340	Laryngeal Squamous Cell Carcinoma (LSCC)	11,388.06	65.83	5,955.13
107	371	GSM303349	Laryngeal Squamous Cell Carcinoma (LSCC)	6,392.77	36.95	1,683.43
108	374	GSM383688	Astrocytoma grade II	7,037.01	40.68	1,989.29
109	375	GSM383690	Astrocytoma grade II	12,815.32	74.08	7,721.21
110	376	GSM383691	Astrocytoma grade II	5,908.06	34.15	866.31
111	377	GSM383692	Astrocytoma grade II	6,331.22	36.60	962.64
112	378	GSM383693	Astrocytoma grade II	9,337.33	53.97	3,788.43
113	379	GSM383696	Astrocytoma grade III	5,578.55	32.25	1,419.63
114	380	GSM383697	Astrocytoma grade III	7,223.15	41.75	1,607.20
115	381	GSM383698	Astrocytoma grade III	6,078.44	35.14	900.17

ID [a]	Primary ID [b]	SAGE Library	Sample	Sum [c]	Average [d]	Max [e]
116	382	GSM383699	Astrocytoma grade III	6,674.31	38.58	1,447.56
117	383	GSM383700	Astrocytoma grade III	6,147.01	35.53	901.03
118	384	GSM383702	Astrocytoma grade III	6,554.15	37.89	1,329.04
119	385	GSM383703	Astrocytoma grade III	7,074.76	40.89	1,375.36
120	386	GSM383709	Ependymoma	8,991.56	51.97	2,914.26
121	387	GSM383710	Ependymoma	17,524.27	101.30	10,222.49
122	388	GSM383713	Ependymoma	7,598.78	43.92	1,180.53
123	389	GSM383717	Ependymoma	13,102.87	75.74	6,906.34
124	390	GSM383720	Glioblastoma multiforme	9,440.19	54.57	4,381.74
125	391	GSM383722	Glioblastoma multiforme	7,891.18	45.61	1,705.33
126	392	GSM383723	Glioblastoma multiforme	4,256.96	24.61	405.42
127	393	GSM383724	Glioblastoma multiforme	7,144.79	41.30	2,959.18
128	394	GSM383726	Glioblastoma multiforme, pooled sample	7,595.79	43.91	2,342.63
129	395	GSM383727	Glioblastoma multiforme	7,108.63	41.09	1,475.19
130	404	GSM383753	Medulloblastoma	3,987.16	23.05	1,069.73
131	410	GSM383767	Benign meningioma	7,090.15	40.98	2,628.12
132	411	GSM383768	Benign meningioma	10,485.53	60.61	3,293.87
133	412	GSM383769	Malignant meningioma	6,528.97	37.74	780.19
134	413	GSM383770	Malignant meningioma	13,488.81	77.97	5,819.14
135	414	GSM383771	Malignant meningioma	11,742.27	67.87	6,540.21

ID [a]	Primary ID [b]	SAGE Library	Sample	Sum [c]	Average [d]	Max [e]
136	418	GSM383781	Oligodendroglioma	8,619.45	49.82	2,944.98
137	419	GSM383783	Breast carcinoma myoepithelium	8,625.17	49.86	2,464.33
138	421	GSM383785	Invasive breast cancer myofibroblasts	5,000.95	28.91	596.32
139	422	GSM383786	Invasive breast cancer myofibroblasts	10,065.79	58.18	4,638.47
140	425	GSM383790	Mammary gland, ductal carcinoma	20,417.00	118.02	15,536.62
141	426	GSM383791	Mammary gland, ductal carcinoma in situ	4,655.57	26.91	414.27
142	427	GSM383793	Mammary gland, ductal carcinoma in situ	29,781.50	172.15	25,275.20
143	428	GSM383794	Mammary gland, ductal carcinoma in situ	23,570.83	136.25	18,977.90
144	429	GSM383796	Mammary gland, ductal carcinoma	10,631.64	61.45	4,752.97
145	430	GSM383797	Mammary gland, ductal carcinoma	27,222.30	157.35	22,422.27
146	440	GSM383807	Mammary gland, ductal carcinoma in situ	20,570.15	118.90	16,071.37
147	441	GSM383808	Mammary gland, ductal carcinoma in situ	11,014.29	63.67	4,792.61
148	442	GSM383811	Breast carcinoma	7,040.24	40.70	1,383.98
149	443	GSM383812	Mammary gland, ductal carcinoma	20,036.49	115.82	13,377.93
150	444	GSM383813	Breast carcinoma	13,029.89	75.32	8,622.92
151	445	GSM383814	Mammary gland, ductal carcinoma	4,676.10	27.03	1,423.16
152	446	GSM383815	Mammary gland, ductal carcinoma	9,776.32	56.51	4,974.52
153	447	GSM383816	Breast cancer	8,458.52	48.89	4,009.82
154	448	GSM383817	Mammary gland, lobular/ductal carcinoma	4,192.31	24.23	609.79
155	449	GSM383818	Breast cancer	6,804.41	39.33	3,230.18

ID [a]	Primary ID [b]	SAGE Library	Sample	Sum [c]	Average [d]	Max [e]
156	450	GSM383819	Mammary gland, ductal carcinoma	6,234.25	36.04	833.76
157	451	GSM383820	Mammary gland, ductal carcinoma	6,752.38	39.03	1,646.47
158	452	GSM383821	Breast cancer	13,936.77	80.56	10,608.99
159	453	GSM383823	Breast carcinoma	5,151.90	29.78	841.43
160	454	GSM383825	Breast cancer	13,304.39	76.90	9,381.63
161	456	GSM383828	Breast carcinoma, metastasis	11,608.60	67.10	6,078.32
162	471	GSM383848	Breast cancer	4,844.49	28.00	588.74
163	473	GSM383850	Cartilage chondrosarcoma	5,846.64	33.80	1,763.61
164	482	GSM383859	Colon adenocarcinoma	11,425.29	66.04	7,562.96
165	501	GSM383884	Esophagus adenocarcinoma	7,577.50	43.80	4,066.64
166	506	GSM383892	Gallbladder adenocarcinoma	3,909.95	22.60	651.66
167	507	GSM383893	Gallbladder tubular adenocarcinoma	23,262.07	134.46	20,154.48
168	508	GSM383894	Gallbladder tubular adenocarcinoma	3,643.40	21.06	624.22
169	510	GSM383897	Kidney, transitional cell carcinoma	9,719.69	56.18	3,732.20
170	515	GSM383903	Liver cholangiocarcinoma metastasis	2,736.32	15.82	1,575.46
171	516	GSM383904	Liver adenocarcinoma	6,378.42	36.87	1,091.61
172	520	GSM383908	Lung adenocarcinoma	12,343.63	71.35	6,586.96
173	523	GSM383915	Lymph node, B cell lymphoma	3,166.97	18.31	555.61
174	524	GSM383921	Muscle, rhabdomyosarcoma	12,051.93	69.66	5,830.00
175	526	GSM383926	Ovary, clear cell carcinoma	4,248.47	24.56	643.71

ID [a]	Primary ID [b]	SAGE Library	Sample	Sum [c]	Average [d]	Max [e]
176	536	GSM383940	Bone, chondrosarcoma	11,045.16	63.84	5,624.30
177	546	GSM383970	Retinoblastoma	3,561.82	20.59	384.45
178	550	GSM383994	Stomach adenocarcinoma	5,821.05	33.65	941.64
179	551	GSM383995	Stomach/lower esophagus adenocarcinoma	5,056.34	29.23	951.40
180	552	GSM383996	Gastroesophageal junction adenocarcinoma	5,161.40	29.83	817.65
181	553	GSM383998	Gastroesophageal junction adenocarcinoma	4,141.08	23.94	1,351.60
182	556	GSM384006	Testis, embyronal carcinoma	5,395.71	31.19	1,055.03
183	557	GSM384007	Thyroid, follicular adenoma	5,268.39	30.45	1,565.75
184	558	GSM384008	Thyroid, follicular carcinoma	6,821.66	39.43	2,188.08
185	565	GSM384025	White blood cells, CD45+, isolated from a mammary gland carcinoma	13,734.90	79.39	10,097.48
			Normal			
1	3	GSM571	Retinal pigment epithelium (RPE)	4,957.60	28.66	4,118.91
2	4	GSM572	Peripheral retina	18,781.47	108.56	8,727.76
3	5	GSM573	Peripheral retina	18,817.72	108.77	12,293.99
4	6	GSM574	Central retina (macula)	33,159.24	191.67	26,692.01
5	12	GSM675	Ovary	6,604.07	38.17	943.44
6	13	GSM676	Brain, greater than 95% white matter	14,125.43	81.65	8,388.62
7	14	GSM677	Breast, luminar mammary epithelium	12,564.89	72.63	9,263.94
8	15	GSM678	Pancreas	7,045.97	40.73	2,243.10

ID [a]	Primary ID [b]	SAGE Library	Sample	Sum [c]	Average [d]	Max [e]
9	18	GSM685	Prostate	8,270.07	47.80	1,461.20
10	28	GSM695	Brain, greater than 95% white matter	7,452.78	43.08	1,259.15
11	38	GSM706	Microvascular endothelial cells (HMVEC)	10,944.38	63.26	4,595.12
12	40	GSM708	Kidney	14,621.15	84.52	11,380.01
13	44	GSM713	Brain, thalamus	11,710.86	67.69	5,649.25
14	49	GSM719	Ovary, surface epithelium	14,663.06	84.76	9,871.75
15	50	GSM721	Pancreas, duct epithelial cells	5,337.64	30.85	993.05
16	56	GSM729	Colonic epithelium	11,119.18	64.27	7,930.74
17	65	GSM738	Peritoneum, sheet (>90% mesothelial cells)	10,930.78	63.18	7,206.91
18	79	GSM760	Mammary gland, ductal epithelium	12,315.07	71.19	7,458.14
19	82	GSM780	Breast tissue (gestational hyperplasia)	11,122.36	64.29	6,977.19
20	83	GSM781	Breast, mammary myoepithelial cells	6,777.11	39.17	3,457.01
21	84	GSM784	Gastric epithelial tissues	2,970.06	17.17	673.21
22	85	GSM786	Frontal cortex	9,488.20	54.85	2,526.75
23	87	GSM819	Muscle, old	7,186.23	41.54	1,708.35
24	88	GSM824	Muscle, young	6,385.15	36.91	2,041.76
25	98	GSM1499	Heart	11,728.64	67.80	6,820.91
26	107	GSM1734	Pooled organs and tissues include: B-lymphocyte (plasmacytoma, mycloma), mammary gland (adenocarcinoma), liver (hepatoblastoma), cervix (adenocarcinoma), testis (embryonal	6,342.94	36.66	1,334.34

ID [a]	Primary ID [b]	SAGE Library	Sample	Sum [c]	Average [d]	Max [e]
			carcinoma), brain (glioblastoma), melanoma, liposarcoma, macrophage (histiocytic lymphoma, histocyte), and T-lymphoblast (lymphoblastic leukemia) // Universal reference			
27	110	GSM2386	Spinal cord	6,358.81	36.76	1,268.14
28	114	GSM2455	Uterine cervix	5,602.43	32.38	889.80
29	118	GSM3242	Skin	6,201.84	35.85	939.67
30	121	GSM3245	CD4+ T-cells	4,088.74	23.63	978.17
31	151	GSM14749	First trimester placenta	14,369.11	83.06	5,960.93
32	152	GSM14750	Full-term placenta	14,962.32	86.49	5,486.47
33	156	GSM14754	Mammary gland, endothelium	7,978.48	46.12	3,535.92
34	157	GSM14755	White blood cells, CD45+CD15+	13,981.86	80.82	9,852.99
35	158	GSM14756	Mammary gland	4,772.65	27.59	1,240.55
36	159	GSM14757	Breast stroma	4,992.33	28.86	810.94
37	177	GSM14777	Thyroid	5,425.39	31.36	1,537.77
38	180	GSM14780	Gastric epithelial tissue from the antrum	4,169.95	24.10	1,615.39
39	183	GSM14784	Bone marrow	3,740.58	21.62	962.65
40	184	GSM14785	Lymph node	5,470.11	31.62	1,897.38
41	188	GSM14789	Bone marrow, CD34+/CD38-/lin- cells	5,811.73	33.59	960.90
42	195	GSM14796	Brain, substantia nigra	12,380.10	71.56	8,363.65
43	197	GSM14798	Liver	7,845.94	45.35	1,162.84

ID [a]	Primary ID [b]	SAGE Library	Sample	Sum [c]	Average [d]	Max [e]
44	198	GSM14799	Fetal brain	8,819.47	50.98	1,508.30
45	199	GSM14801	Breast	4,714.03	27.25	1,216.52
46	200	GSM14802	Bone marrow, CD34+/CD38+/lin+ cells	5,835.05	33.73	1,287.14
47	209	GSM16892	Glomerular endothelial cells	11,206.47	64.78	4,624.66
48	215	GSM37211	Adrenal cortex	10,594.95	61.24	3,595.57
49	221	GSM43175	Bone marrow CD34+ cells	4,352.28	25.16	763.91
50	259	GSM82243	Spermatozoa, pooled sample	3,811.16	22.03	1,429.18
51	260	GSM82458	Hippocampus	17,289.52	99.94	8,268.90
52	261	GSM82459	Spermatozoa	3,814.90	22.05	1,288.15
53	262	GSM85611	Bronchial epithelium	19,653.98	113.61	15,655.95
54	263	GSM85616	Bronchial epithelium	19,665.60	113.67	15,918.74
55	264	GSM85622	Lung parenchyma, pooled sample	8,047.65	46.52	3,531.27
56	265	GSM85623	Lung parenchyma, pooled sample	10,933.13	63.20	7,107.31
57	282	GSM118371	CD14+ monocytes isolated from peripheral blood	7,005.86	40.50	3,953.80
58	283	GSM125335	Bronchial brushings, current smoker	11,183.76	64.65	6,290.86
59	284	GSM125336	Bronchial brushings, current smoker	16,090.33	93.01	11,242.21
60	285	GSM125337	Bronchial brushings, current smoker	17,598.56	101.73	12,699.28
61	286	GSM125338	Bronchial brushings, current smoker	14,418.06	83.34	8,663.11
62	287	GSM125339	Bronchial brushings, current smoker	16,871.13	97.52	10,737.09
63	288	GSM125340	Bronchial brushings, current smoker	12,589.60	72.77	7,447.78

ID [a]	Primary ID [b]	SAGE Library	Sample	Sum [c]	Average [d]	Max [e]
64	289	GSM125341	Bronchial brushings, current smoker	13,698.18	79.18	8,899.54
65	290	GSM125342	Bronchial brushings, current smoker	14,134.83	81.70	9,009.58
66	291	GSM125343	Bronchial brushings, former smoker	17,662.13	102.09	12,756.35
67	292	GSM125344	Bronchial brushings, former smoker	14,756.60	85.30	9,732.40
68	293	GSM125345	Bronchial brushings, former smoker	12,435.45	71.88	7,918.51
69	294	GSM125346	Bronchial brushings, former smoker	12,156.84	70.27	7,015.76
70	295	GSM125347	Bronchial brushings, former smoker	16,306.85	94.26	11,835.62
71	296	GSM125348	Bronchial brushings, former smoker	9,776.02	56.51	4,523.58
72	297	GSM125349	Bronchial brushings, former smoker	17,225.16	99.57	13,193.26
73	298	GSM125350	Bronchial brushings, former smoker	15,298.37	88.43	10,235.55
74	299	GSM125351	Bronchial brushings, former smoker	16,456.57	95.12	11,137.34
75	300	GSM125352	Bronchial brushings, former smoker	29,184.64	168.70	24,955.09
76	301	GSM125353	Bronchial brushings, former smoker	43,563.92	251.81	40,054.67
77	302	GSM125354	Bronchial brushings, former smoker	13,791.23	79.72	8,430.13
78	303	GSM125355	Bronchial brushings, never smoker	14,445.94	83.50	8,827.27
79	304	GSM125356	Bronchial brushings, never smoker	11,234.01	64.94	5,628.80
80	305	GSM125357	Bronchial brushings, never smoker	15,083.77	87.19	10,043.19
81	306	GSM125358	Bronchial brushings, never smoker	19,369.05	111.96	14,172.30
82	307	GSM135388	Skeletal muscle, pretraining young men	1,091.72	6.31	327.52
83	308	GSM135389	Skeletal muscle, 5 days training young men	1,673.61	9.67	435.14

ID [a]	Primary ID [b]	SAGE Library	Sample	Sum [c]	Average [d]	Max [e]
84	309	GSM135390	Skeletal muscle, 6 wk training young men	6,085.48	35.18	1,445.53
85	310	GSM135391	Skeletal muscle, 12 wk training young men	9,198.54	53.17	2,955.42
86	311	GSM135392	Skeletal muscle, detraining young men	1,670.43	9.66	510.41
87	317	GSM136194	Naive CD4 T Cells (CD45RA+)	5,951.44	34.40	1,646.56
88	318	GSM136195	Cord blood-derived activated Th1 cells	3,668.81	21.21	777.29
89	319	GSM136196	Cord blood-derived activated Th2 cells	5,244.54	30.32	1,241.31
90	320	GSM136197	CD56+ NK cells	11,146.54	64.43	6,119.11
91	321	GSM136198	CD8+ T cells	6,216.54	35.93	1,568.84
92	322	GSM136199	CD19+ B cells	7,068.33	40.86	3,270.98
93	324	GSM154137	Hippocampus; control, APOE3/3	8,688.86	50.22	1,959.25
94	328	GSM180670	Lymphocytes from children 1-4 years old (pooled samples)	3,794.27	21.93	843.17
95	342	GSM194647	Oral brushing	8,779.79	50.75	6,217.04
96	343	GSM194648	Oral brushing	7,929.28	45.83	5,434.05
97	344	GSM194649	Oral brushing	3,290.72	19.02	996.85
98	345	GSM194650	Oral biopsy	15,392.44	88.97	8,348.63
99	346	GSM194651	Oral biopsy	27,066.16	156.45	21,312.29
100	347	GSM194652	Oral biopsy	21,279.52	123.00	11,932.33
101	353	GSM296374	Lung biopsy	6,008.93	34.73	2,532.33
102	354	GSM296376	Lung biopsy	7,770.69	44.92	4,270.78
103	355	GSM296377	Lung biopsy	12,084.54	69.85	7,345.68

ID [a]	Primary ID [b]	SAGE Library	Sample	Sum [c]	Average [d]	Max [e]
104	356	GSM296379	Lung biopsy	7,853.75	45.40	3,589.97
105	357	GSM296381	Lung biopsy	4,266.36	24.66	1,166.72
106	358	GSM296382	Lung biopsy	4,420.25	25.55	867.53
107	359	GSM296385	Lung biopsy	4,619.07	26.70	949.28
108	360	GSM296387	Lung biopsy	8,146.47	47.09	3,346.60
109	361	GSM296391	Lung biopsy	17,223.67	99.56	13,839.55
110	362	GSM296392	Lung biopsy	7,750.68	44.80	3,632.41
111	363	GSM296393	Lung biopsy	4,563.77	26.38	944.23
112	364	GSM296644	Lung biopsy	4,274.83	24.71	700.79
113	365	GSM301092	Lung parenchyma	5,875.69	33.96	1,545.29
114	366	GSM301093	Lung parenchyma	6,105.38	35.29	1,612.02
115	372	GSM311354	CD15+ myeloid progenitor cells	3,859.27	22.31	1,108.11
116	373	GSM383686	Fetal cortex astrocytes	10,416.46	60.21	5,373.73
117	415	GSM383772	Cerebellum	6,209.70	35.89	1,015.05
118	416	GSM383775	Cortex, pooled sample	19,926.52	115.18	13,701.49
119	417	GSM383776	Brain, leptomeninges	9,314.33	53.84	1,559.29
120	420	GSM383784	Mammary gland myofibroblasts	6,855.11	39.62	2,167.20
121	457	GSM383831	Mammary epithelium	13,513.78	78.11	9,882.32
122	458	GSM383832	Mammary epithelium, CD24+ cells	6,084.02	35.17	2,197.01
123	459	GSM383833	Mammary epithelium, CD24+ cells	8,297.75	47.96	3,738.81

ID [a]	Primary ID [b]	SAGE Library	Sample	Sum [c]	Average [d]	Max [e]
124	460	GSM383834	Mammary epithelium, CD24+ cells	7,912.32	45.74	3,544.20
125	461	GSM383835	Mammary epithelial stem cells, CD44+	5,910.40	34.16	1,053.51
126	462	GSM383836	Mammary epithelial stem cells, CD44+	5,966.22	34.49	2,013.60
127	463	GSM383837	Mammary epithelium, Muc1+ cells	4,463.04	25.80	990.24
128	464	GSM383838	Mammary epithelium, Muc1+ cells	7,728.04	44.67	3,345.75
129	465	GSM383839	Mammary epithelium	9,813.57	56.73	4,689.67
130	466	GSM383840	Mammary myoepithelium, CD10+ cells	3,364.85	19.45	291.33
131	467	GSM383842	Mammary myoepithelium, CD10+ cells	7,393.51	42.74	2,876.12
132	468	GSM383843	Mammary myoepithelium, CD10+ cells	7,103.37	41.06	2,429.06
133	470	GSM383847	Breast stroma	5,214.63	30.14	654.31
134	489	GSM383869	Colonic epithelium	7,763.19	44.87	4,375.62
135	504	GSM383887	Esophagus	6,359.99	36.76	1,573.47
136	505	GSM383888	Eye lens	9,227.11	53.34	1,526.20
137	509	GSM383895	Gallbladder	19,320.00	111.68	15,346.14
138	512	GSM383900	Kidney glomeruli	6,543.43	37.82	1,410.93
139	513	GSM383901	Kidney	14,856.58	85.88	11,571.46
140	514	GSM383902	Leukocytes	2,991.69	17.29	439.34
141	519	GSM383907	Liver	4,753.54	27.48	1,256.51
142	521	GSM383911	Lung	5,719.58	33.06	1,062.53
143	533	GSM383937	Pancreas	1,813.11	10.48	278.94

ID [a]	Primary ID [b]	SAGE Library	Sample	Sum [c]	Average [d]	Max [e]
144	537	GSM383946	Whole body, fetal	20,274.33	117.19	9,698.99
145	542	GSM383962	Prostate	6,507.12	37.61	1,504.24
146	543	GSM383963	Retina, macula	9,401.41	54.34	3,005.29
147	544	GSM383965	Central retina	5,362.37	31.00	1,130.45
148	545	GSM383968	Peripheral retina	6,866.82	39.69	1,273.68
149	547	GSM383971	Macular retinal pigment epithelium	6,383.72	36.90	1,219.65
150	548	GSM383972	Choroid retinal pigment epithelium	6,133.81	35.46	1,070.63
151	554	GSM384002	Stomach	2,813.83	16.26	654.38
152	555	GSM384003	Stomach	4,489.14	25.95	1,678.31
153	560	GSM384012	Endometrium	5,543.34	32.04	1,695.07
154	562	GSM384017	Vascular endothelium	7,809.21	45.14	3,527.31
155	566	GSM384026	White blood cell, lung macrophage	5,880.42	33.99	2,660.65
156	567	GSM384027	White blood cell, monocyte-depleted mononuclear cells	6,768.19	39.12	2,640.34
157	568	GSM384028	White blood cell, monocyte-depleted mononuclear cells	7,517.19	43.45	3,545.48
158	569	GSM384029	White blood cell, monocyte	6,365.73	36.80	2,362.13
159	570	GSM384030	White blood cell, monocyte	7,059.38	40.81	3,519.80
160	571	GSM384031	White blood cell, monocyte	4,859.38	28.09	1,285.71
161	572	GSM384032	White blood cell, monocyte	5,312.45	30.71	1,613.41
162	573	GSM384033	White blood cell, monocyte	5,880.73	33.99	2,260.41
163	574	GSM384035	White blood cell, plaque macrophage	9,178.68	53.06	6,164.50

ID [a]	Primary ID [b]	SAGE Library	Sample	Sum [c]	Average [d]	Max [e]
164	575	GSM384036	White blood cell, plaque macrophage	6,899.43	39.88	3,380.12
165	576	GSM389906	Total blood, pooled sample	1,511.12	8.73	436.55
166	579	GSM549178	Bone marrow CD34+ cells	4,291.15	24.80	1,158.45
			Cells cultured in vitro			
1	7	GSM668	Kidney, embryonic cell line 293, uninduced cells	4,119.01	23.81	920.45
2	16	GSM680	Prostate carcinoma cell line	14,451.15	83.53	5,428.23
3	17	GSM683	Prostate carcinoma cell line	8,032.90	46.43	1,248.14
4	35	GSM703	Brain, Duke glioblastoma multiforme cell line	7,385.01	42.69	2,224.19
5	41	GSM710	Brain, c-myc negative medulloblastoma cell line mhh-1	12,611.12	72.90	3,597.27
6	42	GSM711	Skin, post-crisis survival fibroblast cell-line	6,721.33	38.85	1,344.27
7	45	GSM714	Ovarian clear cell carcinoma cell line ES-2, poorly differentiated	4,224.37	24.42	635.24
8	47	GSM717	Colon, cell line derived from colorectal carcinoma	5,737.03	33.16	878.79
9	51	GSM722	Ovarian surface epithelium cell line	9,335.95	53.97	3,655.89
10	57	GSM730	Brain, astrocyte cells	10,260.84	59.31	5,293.44
11	59	GSM732	Ovary, carcinoma cell line	6,941.77	40.13	2,725.29
12	71	GSM747	Colon, cancer cell line	3,945.42	22.81	673.61
13	100	GSM1515	PC3 cells	9,711.74	56.14	3,117.03
14	132	GSM9220	Undifferentiated HES3 40P embryonic stem cells	10,187.49	58.89	4,213.98
15	133	GSM9221	Undifferentiated HES4 40P embryonic stem cells	10,184.07	58.87	3,442.32
16	138	GSM14735	Pancreas, epithelium, ductal carcinoma cell line	8,497.95	49.12	4,136.74

ID [a]	Primary ID [b]	SAGE Library	Sample	Sum [c]	Average [d]	Max [e]
17	139	GSM14736	Pancreas, epithelium, ductal carcinoma cell line	7,424.99	42.92	3,797.25
18	154	GSM14752	Prostate epithelium	8,104.98	46.85	4,073.64
19	160	GSM14758	Prostate epithelium, senescent cells	8,132.60	47.01	4,327.05
20	161	GSM14759	Brain, medulloblastoma, cell line	14,106.06	81.54	9,363.64
21	166	GSM14764	Brain, medulloblastoma, cell line	9,439.66	54.56	6,116.90
22	201	GSM14804	Lung cell line	4,493.92	25.98	691.37
23	202	GSM14805	Lung cell line	4,505.79	26.05	806.30
24	205	GSM14916	Neonatal BJ foreskin primary fibroblasts	5,223.66	30.19	696.49
25	207	GSM14918	AT dermal fibroblasts	6,824.56	39.45	2,477.41
26	212	GSM26246	Primary umbilical arterial endothelial cell (HUAEC)	8,496.96	49.12	2,334.75
27	214	GSM32266	Pulmonary microvascular endothelial cells in culture	8,231.22	47.58	3,266.37
28	217	GSM37337	Primary bronchial epithelial cells	3,604.30	20.83	804.84
29	220	GSM41248	Pulmonary microvascular endothelial cells in culture, exposed to sustained low shear stress	10,793.45	62.39	4,304.53
30	222	GSM45608	Pulmonary microvascular endothelial cells in culture, exposed to sustained high shear stress	9,243.96	53.43	3,552.25
31	224	GSM48251	Cultured Schwann cells	9,815.52	56.74	5,439.92
32	228	GSM62240	Primary aortic endothelial cells	7,898.03	45.65	3,049.73
33	229	GSM62241	Primary aortic endothelial cells, exposure to 8h hypoxia	8,534.78	49.33	3,478.05
34	230	GSM62242	Primary aortic endothelial cells, exposure to 24h hypoxia	10,849.78	62.72	6,500.41

ID [a]	Primary ID [b]	SAGE Library	Sample	Sum [c]	Average [d]	Max [e]
35	231	GSM62243	Primary pulmonary artery endothelial cells	11,159.67	64.51	6,360.23
36	232	GSM62244	Primary pulmonary artery endothelial cells, exposure to 8h hypoxia	10,587.00	61.20	4,749.65
37	233	GSM62245	Primary pulmonary artery endothelial cells, exposure to 24h hypoxia	15,119.15	87.39	9,992.40
38	234	GSM66698	HL-60 cells	3,661.81	21.17	920.25
39	235	GSM66712	HL-60 cells exposed to 2.45 GHz radiofrequency for 2h	3,293.77	19.04	982.36
40	236	GSM66714	HL-60 cells exposed to 2.45 GHz radiofrequency for 6h	1,879.82	10.87	639.52
41	266	GSM110378	Barrett long-term primary cell culture	5,547.51	32.07	1,269.91
42	268	GSM110380	Squamous esophagus long-term primary cell culture	4,574.44	26.44	831.72
43	281	GSM113363	Liver stellate cells 15 days	7,912.89	45.74	2,577.13
44	315	GSM136123	Monocyte-derived immature dendritic cells	6,018.65	34.79	2,608.65
45	316	GSM136124	Monocyte-derived mature dendritic cells	5,501.41	31.80	2,514.93
46	351	GSM264073	Resting derived macrophages	4,404.97	25.46	1,030.20
47	396	GSM383730	Glioblastoma multiforme, cell line	5,208.95	30.11	669.20
48	397	GSM383731	Glioblastoma multiforme, cell line	4,908.14	28.37	625.46
49	398	GSM383733	Glioblastoma multiforme, cell line	10,843.73	62.68	5,738.73
50	399	GSM383734	Glioblastoma multiforme, cell line	8,587.36	49.64	2,412.85
51	400	GSM383735	Glioblastoma multiforme, cell line	5,713.49	33.03	1,043.01
52	401	GSM383736	Glioblastoma multiforme, cell line	14,640.95	84.63	5,577.50
53	402	GSM383737	Glioblastoma multiforme, short term culture	6,264.08	36.21	1,501.66
54	403	GSM383738	Glioblastoma multiforme, cell line	10,590.44	61.22	3,409.06

ID [a]	Primary ID [b]	SAGE Library	Sample	Sum [c]	Average [d]	Max [e]
55	405	GSM383759	Medulloblastoma cell line	5,782.01	33.42	2,402.50
56	406	GSM383760	Medulloblastoma cell line	5,253.36	30.37	627.11
57	407	GSM383763	Medulloblastoma cell line	7,391.19	42.72	1,893.42
58	408	GSM383765	Medulloblastoma cell line	7,473.38	43.20	3,351.96
59	409	GSM383766	Medulloblastoma cell line	5,434.11	31.41	606.54
60	431	GSM383798	Breast carcinoma MCF7 cell line	13,279.90	76.76	6,757.32
61	432	GSM383799	Breast carcinoma cell line	12,701.45	73.42	5,328.01
62	433	GSM383800	Breast carcinoma cell line	20,467.40	118.31	12,710.80
63	434	GSM383801	Breast carcinoma cell line	2,977.71	17.21	297.77
64	435	GSM383802	Breast carcinoma cell line	4,349.89	25.14	581.42
65	436	GSM383803	Breast carcinoma cell line	2,988.12	17.27	597.62
66	437	GSM383804	Breast carcinoma cell line	19,247.28	111.26	9,389.55
67	438	GSM383805	Breast carcinoma cell line	15,676.73	90.62	6,646.93
68	439	GSM383806	Breast carcinoma cell line	17,991.89	104.00	8,886.81
69	472	GSM383849	Cartilage chondrosarcoma cell line	4,203.08	24.30	1,088.57
70	474	GSM383851	Cartilage chondrosarcoma cell line	7,612.38	44.00	2,176.87
71	475	GSM383852	Cartilage chondrosarcoma cell line	3,502.94	20.25	515.14
72	476	GSM383853	Cartilage chondrosarcoma cell line	10,719.01	61.96	5,660.93
73	477	GSM383854	Cartilage chondrosarcoma metastasis cell line	4,981.58	28.80	794.93
74	478	GSM383855	Cartilage chondrosarcoma metastasis cell line	7,277.94	42.07	1,898.09

ID [a]	Primary ID [b]	SAGE Library	Sample	Sum [c]	Average [d]	Max [e]
75	479	GSM383856	Cartilage chondrosarcoma metastasis cell line	7,211.56	41.69	1,960.16
76	480	GSM383857	Cartilage chondrosarcoma metastasis cell line	5,016.72	29.00	1,034.76
77	481	GSM383858	Cartilage chondrosarcoma metastasis cell line	4,511.99	26.08	508.87
78	483	GSM383861	Colon adenocarcinoma, cell line	15,700.14	90.75	9,684.51
79	484	GSM383864	Colon adenocarcinoma, cell line	5,448.70	31.50	853.41
80	485	GSM383865	Colon carcinoma, cell line	4,048.02	23.40	404.80
81	486	GSM383866	Colon carcinoma, cell line	3,746.12	21.65	378.40
82	487	GSM383867	Colon carcinoma, cell line	3,961.32	22.90	506.24
83	488	GSM383868	Colon carcinoma, cell line	4,179.31	24.16	422.91
84	490	GSM383872	Embryonic stem cell line BG01	5,770.46	33.36	899.62
85	491	GSM383873	Embryonic stem cell line H1	7,935.64	45.87	2,798.68
86	492	GSM383874	Embryonic stem cell line H1	6,618.33	38.26	1,544.58
87	493	GSM383875	Embryonic stem cell line H13	6,692.61	38.69	954.79
88	494	GSM383876	Embryonic stem cell line H14	6,816.26	39.40	2,344.42
89	495	GSM383877	Embryonic stem cell line H7	6,818.16	39.41	1,611.83
90	496	GSM383878	Embryonic stem cell line H9	6,680.38	38.61	1,150.88
91	497	GSM383879	Embryonic stem cell line H9	6,318.19	36.52	661.00
92	498	GSM383880	Embryonic stem cell line HES3	6,269.09	36.24	995.25
93	499	GSM383881	Embryonic stem cell line HES4	6,551.45	37.87	836.86
94	500	GSM383882	Embryonic stem cell line HSF6	5,556.77	32.12	803.39

ID [a]	Primary ID [b]	SAGE Library	Sample	Sum [c]	Average [d]	Max [e]
95	511	GSM383898	Kidney, embryonic cell line 293, induced cells	6,312.65	36.49	1,535.51
96	517	GSM383905	Liver carcinoma cell line	4,351.40	25.15	566.78
97	518	GSM383906	Liver cholangiocarcinoma cell line	4,313.36	24.93	681.06
98	525	GSM383925	Ovary, carcinoma, cell line	6,892.67	39.84	984.67
99	528	GSM383928	Ovary preneoplasia cell line	5,025.06	29.05	828.74
100	530	GSM383930	Ovary epithelium, short term culture	14,874.89	85.98	10,021.64
101	531	GSM383933	Pancreatic adenocarcinoma, cell line	7,099.93	41.04	2,260.28
102	532	GSM383934	Pancreatic adenocarcinoma, cell line	8,982.04	51.92	1,699.30
103	534	GSM383938	Pancreatic ductal epithelium, short term culture	4,779.34	27.63	775.87
104	535	GSM383939	Pancreatic ductal epithelium, short term culture	5,377.86	31.09	1,000.53
105	538	GSM383948	Prostatic adenocarcinoma, cell line	8,594.31	49.68	2,699.78
106	539	GSM383949	Prostatic adenocarcinoma	6,306.47	36.45	1,387.73
107	540	GSM383952	Prostatic carcinoma cell line	8,137.61	47.04	1,058.55
108	541	GSM383956	Prostatic carcinoma cell line	15,605.88	90.21	6,487.06
109	559	GSM384010	Mixture of cancer cell lines	6,373.02	36.84	1,340.66
110	563	GSM384020	Vascular endothelium, short term culture	9,013.89	52.10	3,808.19
111	580	GSM549179	Bone marrow CD34+ cells cultured for 12 hours under myeloid differentiation stimulus	5,301.58	30.64	1,627.54
112	581	GSM549180	Bone marrow CD34+ cells cultured for 40 hours under myeloid differentiation stimulus	5,839.23	33.75	2,491.19

ID [a]	Primary ID [b]	SAGE Library	Sample	Sum [c]	Average [d]	Max [e]
			Diseased or otherwise altered samples			
1	116	GSM3240	Nodular skin lesion of an AIDS-KS patient	6,099.29	35.26	2,047.62
2	117	GSM3241	Nodular skin lesion of an AIDS-KS patient	5,136.23	29.69	993.43
3	119	GSM3243	AIDS-KS lesion	5,437.97	31.43	2,218.69
4	120	GSM3244	AIDS-KS lesion	4,107.85	23.74	1,245.47
5	146	GSM14744	White blood cells, CD45+CD15+, isolated from a mammary gland ductal carcinoma	9,692.15	56.02	5,775.98
6	150	GSM14748	Vascular endothelium, tumor associated (isolated from mammary gland ductal carcinoma)	7,112.64	41.11	2,268.69
7	216	GSM37212	Adrenal cortex affected by primary pigmented nodular adrenocortical disease	16,221.46	93.77	4,205.56
8	225	GSM52500	Gastric cardia biopt from a metaplastic Barrett's esophagus patient	4,505.03	26.04	1,048.58
9	226	GSM52501	Squamous esophagus biopt from a metaplastic Barrett's esophagus patient	7,915.25	45.75	2,598.74
10	227	GSM52502	Metaplastic Barrett's Esophagus biopt	6,216.02	35.93	1,126.25
11	312	GSM136119	LPS-stimulated CD14+ monocytes	5,284.38	30.55	2,614.38
12	313	GSM136120	M-CSF-induced macrophages	7,965.47	46.04	4,519.94
13	314	GSM136121	GM-CSF-induced macrophages	9,081.10	52.49	5,949.69
14	323	GSM154136	Hippocampus; Alzheimer-affected, APOE3/3	9,438.07	54.56	2,266.67
15	325	GSM154138	Hippocampus; Alzheimer-affected, APOE3/4	6,689.33	38.67	2,747.90

ID [a]	Primary ID [b]	SAGE Library	Sample	Sum [c]	Average [d]	Max [e]
16	326	GSM154139	Hippocampus; Alzheimer-affected, APOE4/4	11,244.55	65.00	6,107.97
17	340	GSM194386	Metaplastic bronchial epithelium	19,376.31	112.00	14,577.13
18	341	GSM194387	Dysplastic bronchial epithelium	13,105.79	75.76	7,320.41
19	350	GSM237078	Endemic Kaposi's sarcoma tissue	6,013.64	34.76	888.61
20	423	GSM383787	Breast stroma, ductal carcinoma in situ associated	3,663.78	21.18	334.67
21	424	GSM383788	Breast stroma, tumor associated	4,779.75	27.63	1,283.45
22	455	GSM383826	Breast juvenile fibroadenoma	9,182.92	53.08	3,391.73
23	502	GSM383885	Esophagus pre-cancer (high grade dysplasia)	11,197.21	64.72	6,565.64
24	503	GSM383886	Esophagus pre-cancer (low grade dysplasia)	12,391.18	71.63	7,130.27
25	522	GSM383914	Lung, tumor associated (focal fibrosis and chronic inflammation)	4,103.62	23.72	606.45
26	549	GSM383973	Synovial fibroblasts from rheumatoid arthritis	7,243.64	41.87	1,934.78
27	561	GSM384016	Vascular endothelium, hemangioma, benign hyperplasia	22,688.19	131.15	12,514.88
28	577	GSM389907	Total blood during EPO treatment, pooled sample	811.75	4.69	162.35
29	578	GSM389908	Total blood after EPO treatment, pooled sample	2,684.90	15.52	747.69

Indexes (GSM#) represent GEO database accession numbers for SAGE libraries (one accession number selected for redundant entries).

[a] ID, Entries are sorted according to GEO database indexes within each Cluster.

[b] Primary ID, listing within a full dataset (See Supplemental Table 1).

[c] Sum, cumulative (total) tag per million (tpm) value for SAGE tags matching established and candidate imprinted genes within the SAGE library.

[d] Average, tpm value for SAGE tags matching established and candidate imprinted genes within the SAGE library.

[e] Max, maximum tpm value for SAGE tags matching established and candidate imprinted genes within the SAGE library.

Particular sum, average and maximum values could be recalculated to the fraction of the total gene expression by dividing tpm value to 1,000,000.

Supplement Table 3. Abundance of SAGE tags (matching established and candidate imprinted genes) within the Clusters.

N	Gene symbol	NlaIII tag	Cancer tissue Cluster (185 catalogues)		Normal tissue and cells Cluster (166 catalogues)		Cells cultured in vitro Cluster (112 catalogues)		Non-tumorous disease tissue and cells Cluster (29 catalogues)	
			Total Tags [a]	Times Detected [b]	Total Tags [a]	Times Detected [b]	Total Tags [a]	Times Detected [b]	Total Tags [a]	Times Detected [b]
1	NDUFA4	TTGGAGATCT	10,681	184	9,226	164	4,905	112	976	29
2	GFI1	TGTACCATAG	18	17	15	11	17	11	6	3
3	NM019610	GCAGATTTAT	62	45	43	36	31	21	8	6
4	**DIRAS3**	CAGAAAAAAA	255	110	184	88	78	48	34	14
5	BMP8B	AGCAAAACTG	58	45	58	46	221	50	7	6
6	FUCA1	CTATTTAGTT	214	97	298	87	48	31	43	18
7	**TP73**	TGGTACCGCC	19	15	39	24	17	10	0	0
8	PRDM16	AGATTGATAT	53	28	50	32	10	6	8	4
9	PEX10	GGAGGCGGCG	117	71	72	48	103	50	8	5
10	WDR8	TCGGTGCAGG	164	91	182	82	203	64	24	14
11	DVL1	GCCCGCAGGG	618	155	574	136	620	101	99	21
12	Q5EBL5	GTTTCCAGGC	8	8	5	5	9	9	1	1
13	TMEM52	TTACACCGGC	27	17	82	29	57	26	4	3
14	HSPA6	TATGAATTTT	200	42	90	33	4	4	29	8
15	PTPN14	ACTTTTTCAA	49,536	181	72,033	159	15,478	110	7,907	29
16	HIST3H2BB	AACTCCTTCG	181	79	125	59	149	57	16	11

N	Gene symbol	NlaIII tag	Cancer tissue Cluster (185 catalogues)		Normal tissue and cells Cluster (166 catalogues)		Cells cultured in vitro Cluster (112 catalogues)		Non-tumorous disease tissue and cells Cluster (29 catalogues)	
			Total Tags [a]	Times Detected [b]	Total Tags [a]	Times Detected [b]	Total Tags [a]	Times Detected [b]	Total Tags [a]	Times Detected [b]
17	OBSCN	CTGAGCGCCG	99	48	154	50	15	9	4	3
18	Q8NGX0	AGAAGGAAAT	4	4	6	6	3	3	1	1
19	VAX2	GGCGATGGGG	14	13	12	11	22	18	2	2
20	OTX1	GCGGTTCCAG	16	12	14	13	18	13	2	2
21	Q96PX6	GCAGATATTC	29	22	37	21	4	4	2	2
22	ABCG8	GGCTCCAAAA	7	6	12	9	1	1	0	0
23	ZFP36L2	TAGAAAGGCA	1,574	160	1,537	148	571	87	190	28
24	CYP1B1	AATGCTTTTA	273	78	481	78	208	41	73	14
25	RPL22	GATGCTGCCA	3,391	181	3,363	166	4,835	112	482	29
26	TIGD1	CGAAAAGCTT	70	52	35	29	30	21	5	5
27	MYEOV2	CAGACTTTTT	934	155	490	129	462	94	66	17
28	FTHFD	TCTGCATCTT	403	68	439	62	5	4	10	6
29	ZIC1	ATAATAGTGG	21	14	5	5	4	4	0	0
30	HES1	CACTATATTT	326	113	353	94	147	52	23	13
31	FGFRL1	AAAGTGCATC	551	117	362	116	256	79	26	12
32	SPON2	TTATGGATCT	1,011	132	873	113	626	45	268	20
33	Q9NYJ6	TTACTGGCCC	90	41	100	34	44	19	2	2

N	Gene symbol	NlaIII tag	Cancer tissue Cluster (185 catalogues)		Normal tissue and cells Cluster (166 catalogues)		Cells cultured in vitro Cluster (112 catalogues)		Non-tumorous disease tissue and cells Cluster (29 catalogues)	
			Total Tags [a]	Times Detected [b]	Total Tags [a]	Times Detected [b]	Total Tags [a]	Times Detected [b]	Total Tags [a]	Times Detected [b]
34	**NAP1L5**	TAGCTTTTAG	152	63	97	35	24	17	19	9
35	DUX2	AAGGGGTGGA	1	1	0	0	2	2	0	0
36	CDH18	ATCGAAACTG	69	20	20	12	2	2	9	2
37	ADAMTS16	TACCCCTGAA	744	167	737	138	195	80	137	25
38	Q8TBP5	ACCCAGCGGG	275	112	621	120	135	56	90	19
39	CSF2	GTGGGAGTGG	62	43	95	53	60	37	16	9
40	BTNL2	GAAGGAAAGA	11	9	12	11	15	11	1	1
41	**FAM50B**	CCTCAGTTTG	125	71	140	68	22	17	16	9
42	C6orf117	GCAAGCTGTT	110	50	89	39	28	19	16	9
43	**HYMAI**	TATATATTGA	16	12	5	5	12	9	0	0
44	**PLAGL1**	ATCATAATGT	38	25	42	33	19	13	6	5
45	**SLC22A2**	AAAATTATAA	13	12	16	13	6	6	3	3
46	**SLC22A3**	TGCGCTAATC	51	30	26	21	39	13	6	3
47	BRP44L	CAGTGTATAT	871	134	989	131	266	67	75	23
48	**DDC**	TGGCTAAATG	224	38	387	41	161	13	22	6
49	**GRB10**	TGCTTTGCTT	241	107	184	74	202	60	20	13
50	GLI3	TAAATACATT	46	34	37	31	21	14	0	0

N	Gene symbol	NlaIII tag	Cancer tissue Cluster (185 catalogues)		Normal tissue and cells Cluster (166 catalogues)		Cells cultured in vitro Cluster (112 catalogues)		Non-tumorous disease tissue and cells Cluster (29 catalogues)	
			Total Tags [a]	Times Detected [b]	Total Tags [a]	Times Detected [b]	Total Tags [a]	Times Detected [b]	Total Tags [a]	Times Detected [b]
51	EVX1	ACGCCCGTGG	0	0	0	0	0	0	0	0
52	HOXA5	AGCCTGTTTA	45	31	23	21	21	19	5	4
53	HOXA2	CATATTTTTT	350	112	377	100	149	43	24	11
54	HOXA3	CTCTTCCTCG	0	0	3	3	2	2	1	1
55	HOXA11	GAGATAGCCC	8	7	4	4	23	13	2	2
56	HOXA4	TGCTAAGAAT	86	42	43	27	25	15	2	2
57	TMEM60	AATCTATCCT	96	52	73	49	109	40	7	6
58	**PEG10**	GAAGTTATAA	562	108	295	69	265	53	15	8
59	MAGI2	TATTAATAGT	159	64	112	46	21	16	3	3
60	**PPP1R9A**	GAAGAGACAA	66	47	65	34	36	21	6	5
61	**SGCE**	TTGGCAGTAT	468	128	263	89	241	67	58	14
62	**TFPI2**	TGCTTTTAAC	133	33	1,442	43	404	41	90	5
63	**MEST**	CTGAATGTAC	351	107	235	61	503	49	22	9
64	**COPG2IT1**	GAGGGATGGC	169	92	215	79	91	46	24	11
65	**CPA4**	TCTGTAAATC	45	32	73	29	91	20	51	5
66	**MESTIT1**	TGTAGTGGTG	10	10	3	3	2	2	4	3
67	**KLF14**	TGGACTCTGG	2	2	3	3	1	1	0	0

N	Gene symbol	NlaIII tag	Cancer tissue Cluster (185 catalogues)		Normal tissue and cells Cluster (166 catalogues)		Cells cultured in vitro Cluster (112 catalogues)		Non-tumorous disease tissue and cells Cluster (29 catalogues)	
			Total Tags [a]	Times Detected [b]	Total Tags [a]	Times Detected [b]	Total Tags [a]	Times Detected [b]	Total Tags [a]	Times Detected [b]
68	SLC4A2	CCCCTCCCTC	623	155	435	129	478	104	97	21
69	FASTK	GGGGGTGGAT	1,493	178	974	151	669	96	163	28
70	PURG	CTGAACAAAG	93	51	39	14	8	7	2	2
71	**DLGAP2**	CCCCAGCCCC	145	75	184	63	611	69	20	8
72	Q8N9I4	CTAAGCGCAG	217	81	313	72	36	23	18	11
73	FAM77D	GTGCCCTACC	4	4	0	0	0	0	1	1
74	GPT	CCAAGTTCAC	76	51	66	36	17	10	12	6
75	**KCNK9**	CCAGGCACTC	21	18	20	16	5	5	6	3
76	LY6D	GAGATAAATG	1,634	33	1,983	51	87	14	612	8
77	APBA1	TGTCTCCTTC	49	39	84	29	13	12	9	5
78	NM182505	TAAAAATAAA	124	71	158	84	120	46	18	11
79	FAM75D1	CCCCACAGGA	1	1	1	1	0	0	1	1
80	**ABCA1**	ATGGGGAGAG	270	126	300	103	107	61	21	14
81	LMX1B	GGAGCCCAGC	36	20	20	14	17	15	2	1
82	EGFL7	GCACAGGCCA	898	155	1,542	112	1,213	95	447	20
83	PHPT1	GCCTATGGTC	1,050	156	690	137	1,192	98	77	23
84	NM144654	GGAAAGATGC	308	98	2,296	72	105	44	16	8

N	Gene symbol	NlaIII tag	Cancer tissue Cluster (185 catalogues)		Normal tissue and cells Cluster (166 catalogues)		Cells cultured in vitro Cluster (112 catalogues)		Non-tumorous disease tissue and cells Cluster (29 catalogues)	
			Total Tags [a]	Times Detected [b]	Total Tags [a]	Times Detected [b]	Total Tags [a]	Times Detected [b]	Total Tags [a]	Times Detected [b]
85	GATA3	AAGGATGCCA	264	51	105	38	201	16	15	7
86	Q9H6Z8	GCAGCAGCCT	14	13	10	7	4	4	0	0
87	LDB1	TCCTGACCAC	142	83	177	87	156	68	27	16
88	**INPP5F V2**	AGATTGAGGC	104	59	97	47	168	26	20	7
89	C10orf93	AACAAAATTA	20	18	17	17	10	7	0	0
90	NKX6-2	ACCGAGAGCC	114	63	121	63	111	55	18	9
91	PAOX	GAGACTCTGT	28	22	40	34	14	14	7	6
92	C10orf91	GGTTCTCAGC	4	4	3	3	2	2	1	1
93	VENTX2	TGCTTTTAAA	162	100	138	74	114	41	15	7
94	**WT1-Alt trans**	CTGGTATATG	19	10	14	4	11	10	0	0
95	**KCNQ1OT1**	AAATATTTAC	10	9	9	9	4	4	1	1
96	**KCNQ1DN**	GGACCCCAAA	27	6	3	3	2	2	1	1
97	**OSBPL5**	GGGGATGGAT	88	50	90	50	56	34	5	4
98	PKP3	AACAGTCAAA	852	74	727	79	213	43	126	12
99	Q8N9U2	ACAAGTATTC	57	31	137	43	27	16	3	3
100	IFITM1	ACCATTGGAT	928	144	593	124	1,209	53	124	19
101	**PHLDA2**	AGCCCGCCGC	239	64	201	58	272	65	47	11

N	Gene symbol	NlaIII tag	Cancer tissue Cluster (185 catalogues)		Normal tissue and cells Cluster (166 catalogues)		Cells cultured in vitro Cluster (112 catalogues)		Non-tumorous disease tissue and cells Cluster (29 catalogues)	
			Total Tags [a]	Times Detected [b]	Total Tags [a]	Times Detected [b]	Total Tags [a]	Times Detected [b]	Total Tags [a]	Times Detected [b]
102	**CDKN1C**	CCCATCTAGC	178	86	261	73	101	37	22	11
103	**SLC22A18**	CTGGGCCTCT	206	96	186	79	122	53	33	16
104	**IGF2/INS**	CTTGGGTTTT	5,813	108	1,784	65	267	26	1,043	16
105	**IGF2AS**	GAGGGCCGTT	6	5	2	2	1	1	2	2
106	**H19**	GCCACCCCCT	953	69	363	45	185	26	226	12
107	**KCNQ1**	GGCAGGAGAC	61	36	121	48	14	12	9	6
108	B4GALNT4	TGGAGCGTCC	256	103	250	43	128	42	13	7
109	RAB1B	TCAGGCATTT	1,734	173	1,659	162	1,199	110	194	28
110	KBTBD3	AAACTACAAA	102	74	105	61	77	35	13	10
111	NTRI	TCCCTCTTCA	61	40	30	18	2	2	6	5
112	ABCC9	TGTCTTTAAA	397	120	422	106	110	52	38	13
113	**RBP5**	CTTCCTGTTA	90	65	129	65	15	11	12	6
114	HOXC4	GTACCTGCTG	56	43	13	12	20	14	4	3
115	HOXC9	TACGGCTCGC	33	27	20	15	38	22	5	4
116	SLC26A10	ACCCTTGAAC	126	55	72	34	24	17	8	6
117	CDK4	GAAGGAAGAA	574	145	227	102	684	96	24	13
118	Q96AV8	TAAACTGATT	96	54	46	32	143	50	6	5

N	Gene symbol	NlaIII tag	Cancer tissue Cluster (185 catalogues)		Normal tissue and cells Cluster (166 catalogues)		Cells cultured in vitro Cluster (112 catalogues)		Non-tumorous disease tissue and cells Cluster (29 catalogues)	
			Total Tags [a]	Times Detected [b]	Total Tags [a]	Times Detected [b]	Total Tags [a]	Times Detected [b]	Total Tags [a]	Times Detected [b]
119	Q9HCM7	TCAATCAGTG	310	109	263	89	45	32	30	17
120	Q8N7V5	ACTCACTGGA	4	4	1	1	2	2	0	0
121	FAM70B	GTGCCTCTGT	9	9	14	11	14	11	4	4
122	FOXG1C	GAACTATATG	115	36	77	10	70	20	9	2
123	PLEKHC1	GTTCAAAGAC	912	144	560	110	600	76	111	17
124	**DLK1**	ATACAGAATA	325	33	792	42	298	19	124	4
125	**MEG3**	TGGGAAGTGG	2,545	124	1,466	95	641	47	311	19
126	RTL1	ACGGCCTGCA	4	4	2	2	2	2	0	0
127	**ATP10A**	GCCCCCAGAG	61	45	34	24	19	12	1	1
128	**PWCR1**	TTGGTGAGGG	141	70	120	69	79	41	24	17
129	**NDN**	ACCTTGCTGG	561	111	346	103	199	53	68	13
130	**SNURF/SNRPN**	CCGCCTCCGG	1,357	158	1,297	140	2,128	83	216	22
131	**MAGEL2**	TAGCATTGTA	33	26	13	12	22	10	2	2
132	**MKRN3**	AAATAATTTA	37	32	35	27	19	14	2	2
133	**UBE3A**	CTGTAAAACA	335	130	337	114	207	69	35	17
134	Q9P168	AGAACTCCAC	4	4	3	3	0	0	1	1
135	SOX8	CAGCGTCTCC	1,216	65	133	32	61	23	23	4

N	Gene symbol	NlaIII tag	Cancer tissue Cluster (185 catalogues)		Normal tissue and cells Cluster (166 catalogues)		Cells cultured in vitro Cluster (112 catalogues)		Non-tumorous disease tissue and cells Cluster (29 catalogues)	
			Total Tags [a]	Times Detected [b]	Total Tags [a]	Times Detected [b]	Total Tags [a]	Times Detected [b]	Total Tags [a]	Times Detected [b]
136	SALL1	ACATTTCTAG	11	11	3	3	4	4	0	0
137	C16orf57	GGATTTTAAT	197	98	172	94	177	56	20	12
138	ACD	CGGCAAAAAA	196	104	116	72	133	58	21	10
139	FOXF1	TTCCTCCTCT	53	40	44	33	48	22	6	5
140	**ANKRD11**	AAAGCTGACA	184	98	120	75	106	54	10	8
141	Q8N206	ACATTCAGAA	3	3	4	4	2	2	0	0
142	TMEM88	CTGGGCTTCG	39	25	73	33	36	10	22	7
143	PYY2	TTCACTCCCG	16	10	6	3	6	5	1	1
144	HOXB3	AACTCAGCTC	146	69	90	50	68	33	12	8
145	HOXB2	AAGCACAAGC	87	42	53	28	61	25	5	5
146	Q8N8L1	GGGTCTGAGG	337	31	754	25	13	13	176	13
147	FAM59A	TGCAGAGAAA	35	26	34	23	22	17	7	6
148	BRUNOL4	GCTGTTCTTG	177	50	149	25	8	8	43	5
149	**TCEB3C**	ACCTCCCAGG	6	6	11	10	6	6	1	1
150	Q8NE65	TTGGTCAGGC	5,685	185	4,808	162	2,414	112	1,031	29
151	Q8NB05	TGCTCGGGAA	1	1	4	4	0	0	0	0
152	PPAP2C	GTGTTCTTGG	244	79	269	64	225	55	30	12

N	Gene symbol	NlaIII tag	Cancer tissue Cluster (185 catalogues)		Normal tissue and cells Cluster (166 catalogues)		Cells cultured in vitro Cluster (112 catalogues)		Non-tumorous disease tissue and cells Cluster (29 catalogues)	
			Total Tags [a]	Times Detected [b]	Total Tags [a]	Times Detected [b]	Total Tags [a]	Times Detected [b]	Total Tags [a]	Times Detected [b]
153	TSH3	TTCTTATTTT	1,531	170	1,830	145	882	99	137	20
154	CHST8	GTTTCCAGAG	79	37	42	27	34	22	5	4
155	ZNF225	TGGTATGTAT	5	5	14	13	3	3	4	3
156	ZNF229	TTGTAACCTC	67	44	43	26	21	21	2	2
157	**ZNF264**	GCTTCAGTGG	222	119	228	94	55	35	35	18
158	**ZIM2/PEG3**	TTTTCACCAT	341	71	214	53	27	17	20	7
159	LILRB4	GGAAAATGGG	149	71	404	67	37	23	48	12
160	ZNF550	AGAAATGTAC	22	18	17	16	22	18	2	2
161	CHMP2A	GGTGATGAGG	804	162	738	144	547	103	89	27
162	ZNF42	GTCAGAACAC	254	114	386	109	114	55	26	17
163	ISM1	AATATTATCA	2	2	1	1	2	2	0	0
164	**NNAT**	CAGTTGTGGT	2,416	65	386	38	138	18	21	3
165	**BLCAP**	CCTGTCCTTT	513	138	476	126	239	76	54	16
166	**L3MBTL**	TGTGTATGTG	107	69	124	60	50	29	19	12
167	**GNAS**	ATTAACAAAG	4,627	179	3,643	164	3,342	110	265	28
168	**GNASAS**	TCCATTAGAA	5	5	4	4	12	8	0	0
169	COL9A3	AAGGAGCGGG	1,176	138	357	113	838	75	38	16

N	Gene symbol	NlaIII tag	Cancer tissue Cluster (185 catalogues)		Normal tissue and cells Cluster (166 catalogues)		Cells cultured in vitro Cluster (112 catalogues)		Non-tumorous disease tissue and cells Cluster (29 catalogues)	
			Total Tags [a]	Times Detected [b]	Total Tags [a]	Times Detected [b]	Total Tags [a]	Times Detected [b]	Total Tags [a]	Times Detected [b]
170	C20orf20	ACCTCACTCT	106	62	99	62	130	62	5	4
171	SIM2	AAGGAAGATT	567	155	590	141	407	87	47	19
172	DGCR6	CAGAAGAGGC	1,304	170	1,260	144	719	99	103	25
173	FLJ20464	CGTGAAATTC	2	2	2	2	1	1	0	0

Entries are sorted according to the established gene location (see Supplemental Table 1).

Bold denotes established imprinted genes (53 in total).

[a] Total tags accounted within the Cluster.

[b] Number of times the tag was detected at least once in a library within the Cluster.
